# Distinct thalamic functional connectivity and volume patterns across focal epilepsies in children: A multimodal neuroimaging study

**DOI:** 10.1002/epi.70109

**Published:** 2026-01-28

**Authors:** Xiyu Feng, Aswin Chari, Maria H. Eriksson, Freya Prentice, Xiaosong He, Hua Xie, Leigh Sepeta, M. Zubair Tahir, Jonathan D. Clayden, Martin M. Tisdall, Torsten Baldeweg, Rory J. Piper

**Affiliations:** ^1^ Developmental Neurosciences, Great Ormond Street Institute of Child Health University College London London UK; ^2^ Department of Neurosurgery, Great Ormond Street Hospital London UK; ^3^ Department of Psychology University of Science and Technology of China Hefei China; ^4^ Children's National Hospital Washington USA; ^5^ Developmental Imaging and Biophysics Unit, Great Ormond Street Institute of Child Health University College London London UK

**Keywords:** atrophy, focal epilepsy, functional connectivity, pediatric, thalamus

## Abstract

**Objective:**

The thalamus is a key hub in seizure propagation, and its nuclei are emerging targets for neuromodulation. However, the contributions of individual nuclei to epileptic networks remain unclear, particularly in children, who are less studied than adults. We investigated structural and functional thalamic alterations across different pediatric focal epilepsies and their associations with clinical features and postsurgical outcomes.

**Methods:**

We retrospectively studied children with temporal lobe epilepsy (TLE), frontal lobe epilepsy (FLE), and posterior quadrant epilepsy (PQE) and healthy controls. The thalamus was segmented into four nuclei groups (anterior, lateral, medial, pulvinar) using the THOMAS pipeline on T1‐weighted magnetic resonance imaging (MRI) to estimate volumes. Functional connectivity was assessed with functional MRI using node strength, capturing total thalamic connectivity across the brain. We compared patients with controls and evaluated associations with hippocampal sclerosis, history of focal to bilateral tonic–clonic seizures, and postsurgical seizure freedom.

**Results:**

Among 136 children with focal epilepsy (81 TLE, 36 FLE, 19 PQE; mean age = 13.0 years) and 70 controls (mean age = 13.4 years), ipsilateral thalamic volume reductions were observed in the following: anterior and lateral nuclei and pulvinar in TLE, anterior and lateral nuclei in FLE, and pulvinar in PQE (Cohen *d* = .52–.70, all Bonferroni‐corrected *p* < .05). In contrast, medial nuclei volume increase was associated with history of seizure generalization (partial η^2^ = .06). Functional connectivity was bilaterally reduced across epilepsy groups (partial η^2^ = .03), most consistently in the pulvinar (Cohen *d* = .25–.68). Within TLE, hippocampal sclerosis was associated with increased anterior nucleus connectivity (partial η^2^ = .17), distinguishing it from other pathologies.

**Significance:**

We demonstrate both shared and syndrome‐specific thalamic abnormalities in pediatric focal epilepsy. Common patterns included ipsilateral thalamic volume loss, indicating cumulative disease burden, and reduced bilateral functional connectivity, particularly in the pulvinar, likely reflecting thalamocortical decoupling. These findings advance understanding of seizure networks beyond the epileptogenic zone and provide a foundation for personalized thalamic‐targeted neuromodulation strategies.

**Key points**

- Ipsilateral thalamic volume loss and reduced bilateral functional connectivity are common across pediatric focal epilepsies.
- Pulvinar shows consistent functional connectivity reductions.
- Medial nuclei enlargement is linked to seizure generalization.
- TLE with hippocampal sclerosis shows increased anterior nucleus functional connectivity.
- Findings implicate suppression of widespread thalamocortical networks.

## 1 INTRODUCTION

The thalamus is a key structure in seizure propagation^1^, ^2^ and a target for neuromodulation^3^ in focal and generalized onset epilepsy. Although stereo‐electroencephalography (stereo‐EEG) has long been used to delineate epileptogenic networks, criteria for implanting thalamic electrodes is not well defined,^4^ particularly in children, where the risk of invasive sampling must be carefully balanced against clinical benefits. Given the variable efficacy of thalamic neuromodulation and the limited pediatric data, noninvasive studies are needed to understand thalamic involvement in epilepsy and guide thalamic neuromodulation.

Network neuroscience conceptualizes epilepsy as a disorder of disrupted brain connectivity.^5^ Functional magnetic resonance imaging (fMRI) noninvasively assesses functional connectivity (FC), with hubness metrics such as node strength identifying highly connected regions central to brain networks.^6^ These hubs, although essential for efficient information flow, may also facilitate seizure spread.^7^ In adults with temporal lobe epilepsy (TLE), increased thalamic hubness has been associated with focal to bilateral tonic–clonic seizures (FBTCS)^2^ and postoperative seizure recurrence.^8^ Furthermore, structural changes often accompany functional alterations^1^; thalamic volume loss is common in TLE^9^ and correlates with poorer surgical outcomes,^10^ suggesting that combined structural–functional assessment may provide a more complete understanding of thalamic influence on seizure propagation mechanisms and surgical outcomes.

Individual thalamic nuclei may contribute differently to epilepsy syndromes. The anteroventral nucleus, structurally connected to hippocampus and cingulate cortex, is the only thalamic deep brain stimulation (DBS) target approved for adults with drug‐resistant epilepsy, with the most consistent benefits in TLE and frontal lobe epilepsy (FLE).^3^, ^11^ The pulvinar shows promise in posterior quadrant epilepsy (PQE) and select TLE cases.^11^, ^12^, ^13^, ^14^ The medial pulvinar connects with mesial temporal and prefrontal cortices and is often engaged early in temporal‐onset seizures,^15^ whereas the lateral pulvinar primarily connects with occipital and parietal cortices. The centromedian nucleus is also under investigation for generalized epilepsies.^16^, ^17^ Connectivity patterns of the targeted thalamic region may predict stimulation response,^18^, ^19^ although precise markers remain to be determined.

Despite advances, no study has comprehensively examined nuclei‐specific thalamic changes across epilepsy syndromes with different seizure onset locations and pathologies. A further gap is the lack of pediatric data.^20^ Building on our prior work, which demonstrated macroscale thalamic connectivity gradients in pediatric TLE and revealed varying influences along the axes across the whole thalamus,^21^ the present study addresses the need to extend from global thalamus gradients to a fine‐grained anatomical parcellation of nuclei that more closely aligns with clinical intervention targets. Using a large multimodal MRI dataset of 136 children with focal epilepsy, we assessed thalamic nuclei volumes and FC‐based node strength, compared children with TLE, FLE, and PQE to healthy controls, and examined associations with FBTCS history, the presence of hippocampal sclerosis (HS) within TLE, and postsurgical seizure outcomes.

## 2 MATERIALS AND METHODS

**FIGURE 1:**
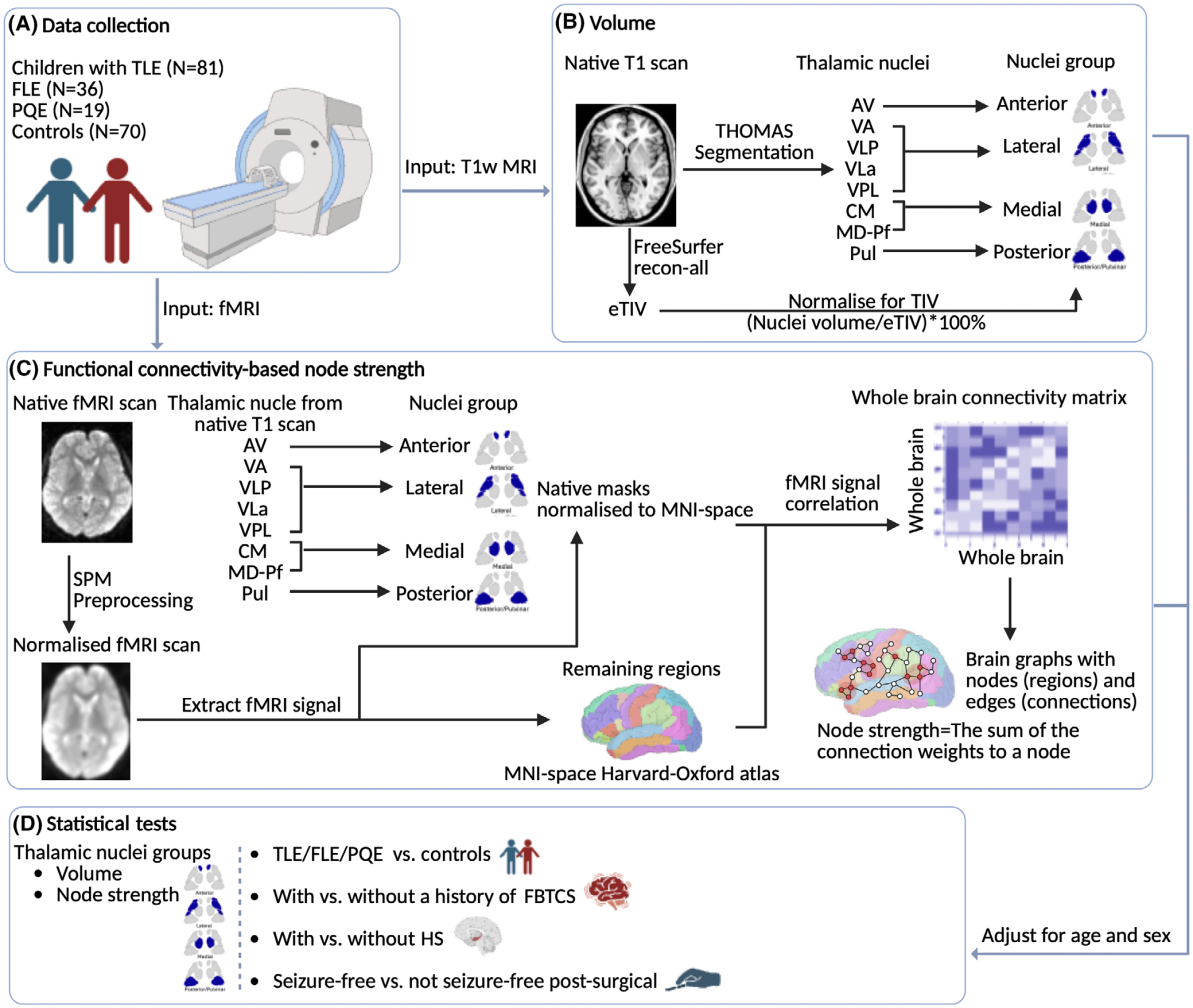
The workflow of this study. AV: anteroventral nucleus; CM, centromedian nucleus; eTIV, estimated total intracranial volume; FBTCS, focal to bilateral tonic–clonic seizures; FLE, frontal lobe epilepsy; fMRI, functional magnetic resonance imaging; HS, hippocampal sclerosis; MD‐Pf, mediodorsal nucleus; MNI, Montreal Neurological Institute; PQE, posterior quadrant epilepsy; Pul, pulvinar; SPM, Statistical Parametric Mapping; T1w, T1‐weighted; TLE, temporal lobe epilepsy; VA: ventral anterior nucleus; VLa, ventral lateral anterior nucleus; VLP, ventral lateral posterior nucleus; VPL, ventral posterolateral nucleus.

Figure 1 shows the workflow.

### 2.1 Participants

We retrospectively identified children with unilateral focal epilepsy from consecutive pediatric candidates undergoing evaluation for epilepsy surgery at Great Ormond Street Hospital, London, UK between 2005 and 2023. Diagnoses were determined by a multidisciplinary evaluation including neurological history and examination, EEG, MRI, positron emission tomography (PET), and neuropsychological tests. Patients were classified according to the putative seizure onset zone as temporal lobe, frontal lobe, or posterior quadrant epilepsy. The control group included typical developing children scanned at the same institution using an identical protocol (see Northam et al.^22^, ^23^ for full details of the recruitment). Controls were required to have the following: (1) no psychological, neurodevelopmental, or neurological diagnoses; (2) normal MRI findings; (3) no chronic medical conditions, past hospitalizations, major perinatal complications, or significant injuries; and (4) age‐appropriate developmental milestones, with attendance at age‐appropriate grade with no special education placement. Participants were excluded if (1) large lesions were seen on the T1 scan that deformed brain architecture, preventing accurate parcellation; (2) there was poor image quality, such as severe fMRI signal dropout; or (3) there was excessive scanner motion.

Ethical approval was granted by the Great Ormond Street Hospital and UCL Great Ormond Street Institute for Child Health (22NP03).

### 2.2 MRI acquisition

Participants were scanned using either a 1.5‐T Siemens Avanto scanner (2005–2018, *n* = 103 patients, *n* = 70 controls) or a 3‐T Prisma scanner (from 2018 onward, *n* = 33 patients). ComBat^24^ harmonization method was applied to remove scanner effects. We evaluated the ComBat harmonization effect (Figure S3) and confirmed that variation of scanner strength in data was removed.

Three‐dimensional T1‐weighted images were acquired using a fast low‐angle shot gradient echo sequence (slice thickness = 1 mm). fMRI echo planar imaging was performed using the following protocols: (1) 3‐mm slice thickness and echo time (TE)/repetition time (TR) = 50/3320 ms (2005–2013), (2) 3‐mm slice thickness and TE/TR = 30/2160 ms (2013–2018), and (3) 2.5‐mm slice thickness and TE/TR 26/1250 ms (from 2018 onward). All participants underwent a verb generation fMRI paradigm as previously described by Croft et al.^25^

### 2.3 Structural imaging processing: Volume estimation

Thalamic nuclei volumes were estimated from native T1‐weighted scans using the THOMAS pipeline.^26^ Volumes were adjusted for estimated total intracranial volume derived via FreeSurfer. Scanner‐related variability was adjusted using ComBat.^24^ For consistency with the connectivity analysis, nuclei were grouped into four regions: anterior, lateral, medial, and posterior (see Section 2.4.2). Volumes were further adjusted for age and sex using generalized linear models (GLMs) and *Z*‐scored relative to the control group. *Z*‐scoring was performed separately for each hemisphere to account for natural anatomical asymmetries, and patient values were classified as ipsilateral or contralateral to the seizure focus.

### 2.4 Functional imaging processing

#### 2.4.1 fMRI preprocessing

fMRI preprocessing was performed using the CONN 22.a^27^ and SPM 12^28^ standard pipeline, including realignment with correction of susceptibility distortion interactions, slice timing correction, outlier detection, direct segmentation and normalization, and denoising. Briefly, all scans were realigned to a reference image and resampled to correct for motion and magnetic susceptibility interactions. Temporal misalignment between different slices of the functional data was corrected. Outlier frames were identified based on framewise displacement (FD; >2 mm) or global blood oxygen level‐dependent signal deviations (>9 SD).^29^, ^30^ Participants with excessive motion (mean FD > 1.5 mm) were excluded. Functional and anatomical data were normalized into standard Montreal Neurological Institute space, segmented into gray matter, white matter, and cerebrospinal fluid tissue classes, and resampled to 2‐mm isotropic voxels following a direct normalization procedure. Last, functional data were denoised by regressing the following confounds: motion, physiological (white matter and cerebrospinal fluid), task design matrix (to remove task effect), outlier frame (according to the number of outliers identified for each session), and linear trends. A high‐pass filter of .008 Hz was applied.

#### 2.4.2 Connectivity matrix generation

We generated whole‐brain FC matrices by calculating Pearson correlation coefficients between preprocessed fMRI time series, including two sets of brain regions. (1) Thalamic nuclei segmentation was estimated in each participant's native T1 scan image using the THOMAS^26^ pipeline. Due to the coarse spatial resolution of fMRI and high signal correlation across nearby voxels, we followed previous studies^31^, ^32^ in grouping thalamic nuclei into four anatomical subdivisions: “anterior” (anteroventral nuclei), “lateral” (ventral anterior, ventral lateral anterior, ventral lateral posterior, and ventral posterolateral nuclei), “medial” (centromedian and mediodorsal nuclei), and “posterior” (pulvinar). Despite its small size, we retained the anteroventral nucleus as a distinct group, given its clinical relevance as a DBS target and our specific interest in it regarding HS, TLE, and FBTCS. (2) The remaining 104 cortical and subcortical regions were defined using the Harvard–Oxford atlas.^33^ Scanner‐related variability in the connectivity matrices was harmonized using ComBat.^24^

### 2.5 FC analysis

#### 2.5.1 Node strength

For computing node strength, we generated whole‐brain FC networks following the previous methodology.^8^ Starting with a graph that connected all 122 brain regions using 121 edges (i.e., connections), we added edges in descending order of edge magnitude (negative and positive correlations of the same magnitude were treated as equal in rank, e.g., −.5 ranked the same as +.5). This generated a series of networks at densities ranging from 5% to 50% in 1% increments, including both positive and negative correlations.

Node strength (the sum of strength of connections to a region) was calculated for each thalamic subdivision using the Brain Connectivity Toolbox.^34^ We computed the area under the curve (AUC) for node strength across the 5%–50% range of network densities.^8^ The AUC values were adjusted for age and sex using GLMs and *Z*‐scored relative to the control group. *Z*‐normalization was performed separately for each hemisphere to account for intrinsic brain functional asymmetries (a patient's nuclei on the right side were *Z*‐scored to the corresponding right‐sided nuclei in the control group, and vice versa), and then for patients, the values were classified as ipsilateral or contralateral to the seizure focus (instead of left and right hemispheric) to investigate the laterality effect of seizure onset. *Z*‐scored node strength was used for subsequent statistical analysis.

#### 2.5.2 Edgewise connectivity profiles

To complement the node strength analysis, we examined the individual functional connections (edges) between each thalamic nuclei group and all other brain regions. This analysis was for two purposes: (1) to illustrate typical connectivity profiles in healthy controls, capturing the functional specificity of each thalamic subdivision; and (2) to explore how alterations in thalamocortical and thalamosubcortical connectivity may underlie the observed node strength differences in the epilepsy groups.

Connectivity values for each edge were extracted from the whole‐brain connectivity matrices. The β coefficients from a GLM were used to adjust the connectivity values accounting for age and sex. In the control group, values were averaged across hemispheres for each nucleus. In patients, connectivity values were grouped as ipsilateral or contralateral to the seizure focus. Independent *t*‐tests were conducted to identify patients' deviations from the controls' pattern. Results were visualized using the Simple Brain Plot.^35^

### 2.6 Statistics

Statistical analyses were performed in SPSS (IBM SPSS Statistics, version 29).

To examine overall differences in node strength and nuclei volume between children with focal epilepsy and healthy controls, we used a mixed‐model analysis of variance (ANOVA). This model included group (control vs. overall focal epilepsy with TLE, FLE, and PQE combined) as a between‐subjects factor and thalamic nucleus (anterior, lateral, medial, and pulvinar of each hemisphere) as a within‐subject factor. Laterality (ipsilateral vs. contralateral) was not included in this model because the control group does not have a defined seizure focus for laterality.

We then performed a three‐way mixed‐model ANOVA to compare epilepsy subtypes. This model included group (TLE, FLE, PQE) as a between‐subjects factor, and thalamic nucleus and laterality (ipsi‐ and contralateral) as within‐subject factors.

To assess deviations from control values, post hoc one‐sample *t*‐tests were planned to compare each epilepsy group (overall focal epilepsy, TLE, FLE, PQE) against zero (reflecting the control mean) for each thalamic nucleus on both ipsilateral and contralateral sides. Paired *t*‐tests were used within patient groups to assess laterality effects.

We further examined the impact of clinical subgroups using three‐way mixed‐model ANOVAs, including group as a between‐subject factor (patients with vs. without a history of FBTCS; with vs. without a history of status epilepticus; congenital vs. acquired etiology; TLE with vs. without HS; postsurgically seizure‐free vs. not seizure‐free), and nuclei and laterality as within‐subject factors.

Associations between structural and functional features were examined using Pearson correlation analyses, including (1) volume versus node strength and (2) their relationships with epilepsy duration.

Multiple comparison correction was applied using the Bonferroni method where appropriate. A corrected *p*‐value of <.05 was considered statistically significant. Effect sizes were reported to highlight the importance of discriminative power, in line with recommendations for biomarker evaluation in epilepsy research.^36^ A moderate to large effect size is traditionally defined as Cohen *d* > .5^37^ and partial η^2^ > .06.

## 3 RESULTS

Table 1 summarizes demographic and clinical characteristics. We analyzed data from 81 children with TLE, 36 with FLE, 19 with PQE, and 70 typically developing controls. Eighty‐six patients had documented seizure outcomes after resection surgery at final clinical follow‐up (years of follow‐up for seizure‐free patients: median = 1.2, interquartile range [IQR] = 1.0–3.3; not seizure‐free: median = 1.2, IQR = 1.0–3.7). Table S7 shows the number of antiseizure medications used at the time of scanning (median = 2) and the total number trialed (median = 3). Fifteen participants with large lesions preventing accurate parcellation of brain regions, four with bad image quality, and seven with excessive motion were excluded.

**TABLE 1:** Demographic, clinical, and neuroradiological characteristics of participants.

Characteristic	TLE, *n* = 81	FLE, *n* = 36	PQE, *n* = 19	Control, *n* = 70
Age at scan, years, mean (SD)	13.1 (3.0)	11.8 (3.2)	13.2 (3.1)	13.4 (3.1)
Sex, F:M ratio	46:35	19:17	11:8	34:36
Side of seizure focus, L:R ratio	62:19	27:9	13:6	‐
Etiological categories				
Hippocampal sclerosis	28	‐	‐	‐
Focal cortical dysplasia	11	15	3	‐
Long‐term epilepsy‐associated tumor	29	9	7	‐
Vascular	3	2	4	‐
Scarring	3	1	3	‐
Mixed, other, or undetermined	7	9	2	‐
Duration of epilepsy, years, mean (SD)	6.5 (4.1)	5.8 (3.8)	7.6 (4.0)	‐
History of focal to bilateral tonic–clonic seizures				
Yes	29	8	8	‐
No	46	27	11	‐
No information	6	1	0	‐
History of status epilepticus				
Yes	21	8	4	‐
No	60	27	15	‐
No information	0	1	0	‐
Seizure freedom after surgery, *n* (years of follow‐up, median [IQR])				
Yes	40 (1.0 [1.0–1.6])	14 (2.3 [1.2–5.0])	4 (2.6 [1.5–5.2])	‐
No	13 (1.0 [1.0–1.9])	11 (2.1 [1.3–4.5])	4 (2.6 [1.6–7.3])	‐
No outcomes or no surgery	28	11	11	‐

*Note*: Seizure‐free is equivalent to an Engel IA/International League Against Epilepsy 1 outcome. Abbreviations: F, female; FLE, frontal lobe epilepsy; IQR, interquartile range; L, left; M, male; PQE, posterior quadrant epilepsy; R, right; TLE, temporal lobe epilepsy.

### 3.1 Thalamic FC and volumes in children with focal epilepsies

#### 3.1.1 Overall focal epilepsy group‐level patterns

**FIGURE 2:**
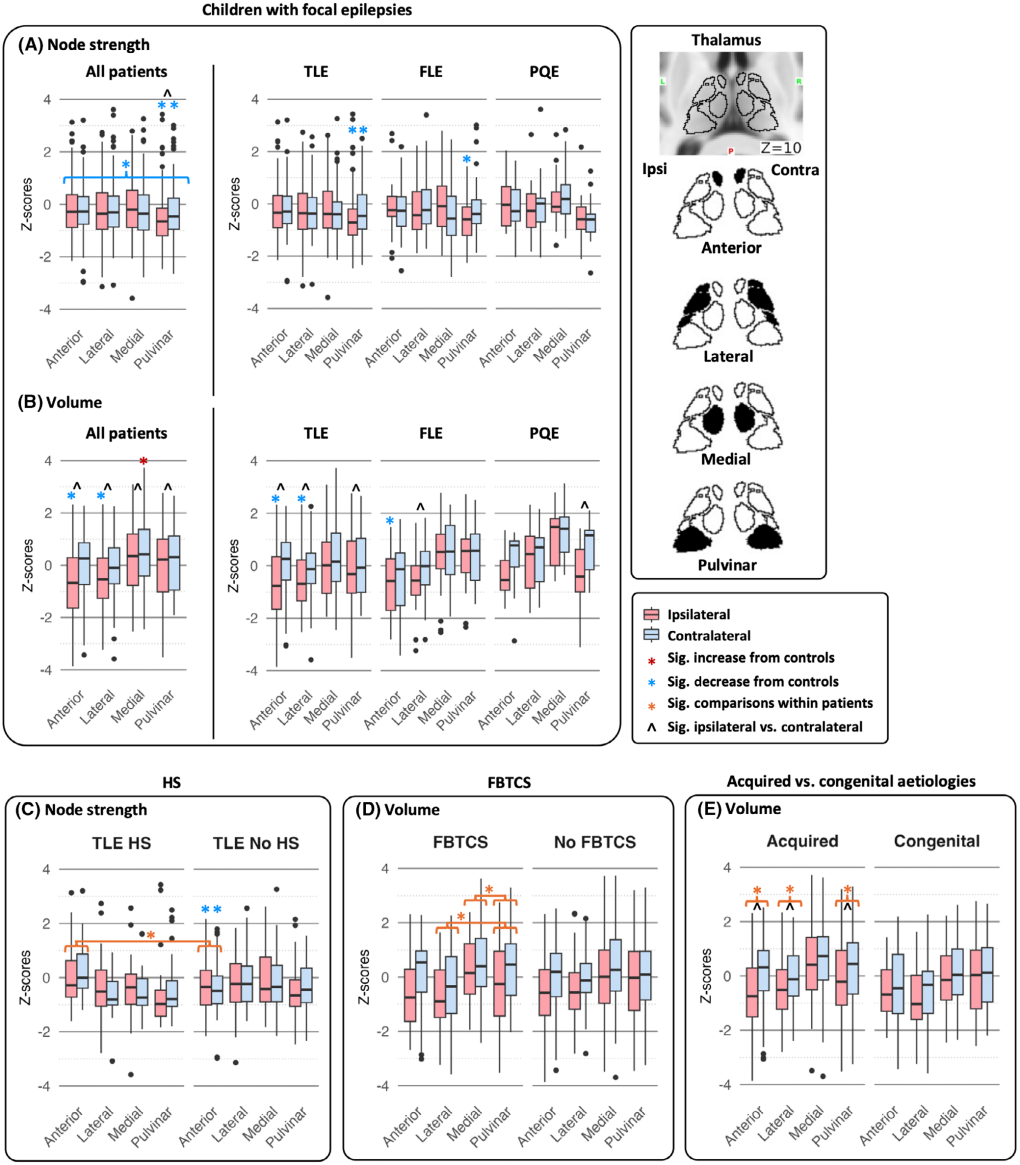
Group differences in thalamic (A) node strength and (B) volume in children with temporal (TLE), frontal (FLE), and posterior quadrant epilepsy (PQE). (C) Associations between thalamic node strength and the presence of hippocampal sclerosis (HS). (D) Associations between thalamic volume and a history of focal to bilateral tonic–clonic seizures (FBTCS). (E) Associations between thalamic volume and acquired etiologies. (A) Node strength and (B) volume alterations are shown for each epilepsy group compared to healthy controls, and for the ipsilateral versus contralateral sides within patient groups. (C) Node strength of anterior nucleus was elevated bilaterally in TLE with HS compared to those without HS. (D) Children with FBTCS history showed increased volume in the medial nuclei and reduced volume in the lateral nuclei, relative to the pulvinar. These patterns were not observed in those who had never experienced FBTCS. In other words, FBTCS was marked by reduced lateral and increased medial thalamic volumes. (E) Patients with acquired etiologies showed ipsilateral volume loss relative to the contralateral side, whereas patients with congenital etiologies did not show this lateralized difference. Thalamic subdivisions include anterior (anteroventral nucleus), lateral (ventral anterior, ventral lateral anterior/posterior, and ventral posterolateral nuclei), medial (centromedian and mediodorsal nuclei), and pulvinar. Box charts present the median of data. *Significant (Sig.) at Bonferroni‐corrected *p* < .05.

Children with focal epilepsy showed reduced node strength across thalamic nuclei bilaterally compared to controls (main effect of group: *p* = .013, partial η^2^ = .03; Table 2, Figure 2). No significant main effect of nucleus or group × nucleus interaction was observed (*p* > .05). To explore nucleus‐specific differences in more detail, we conducted post hoc comparisons between patients and the control mean (zero). These analyses revealed that the pulvinar had the most pronounced reduction in node strength (ipsilateral: corrected *p* < .001, Cohen *d* = .55; contralateral: corrected *p* = .032, Cohen *d* = .25; ipsilateral < contralateral: corrected *p* = .024, Cohen *d* = .26; Table 3).

**TABLE 2:** Mixed analyses of variance examining effects of group, nuclei, and laterality.

Group	Effect	Node strength			Volume		
		*F*(df1, df2)	Sig.	Partial η^2^	*F*(df1, df2)	Sig.	Partial η^2^
Overall focal epilepsy vs. controls	Nuclei	1.27 (7, 198)	.268	.04	7.72 (7, 198)	<.001^a^	.21
	Nuclei × group	1.27 (7, 198)	.268	.04	7.72 (7, 198)	<.001^a^	.21
	Group	6.30 (1, 204)	.013^a^	.03	.06 (1, 204)	.802	.00
TLE vs. FLE vs. PQE	Nuclei	4.25 (3, 131)	.007^a^	.09	19.42 (3, 131)	<.001^a^	.31
	Nuclei × group	.83 (6, 264)	.544	.02	2.74 (6, 264)	.013^a^	.06
	Laterality	.47 (1, 133)	.492	.00	45.17 (1, 133)	<.001^a^	.25
	Laterality × group	.03 (2, 133)	.975	.00	3.62 (2, 133)	.029^a^	.05
	Nuclei × laterality	2.78 (3, 131)	.043^a^	.06	2.12 (3, 131)	.101	.05
	nuclei × laterality × group	1.05 (6, 264)	.396	.02	4.16 (6, 264)	.001^a^	.09
	Group	.63 (2, 133)	.535	.01	.07 (2, 133)	.933	.00
TLE‐HS vs. TLE‐other	Nuclei	2.28 (3, 77)	.086	.08	12.06 (3, 77)	<.001^a^	.32
	Nuclei × group	5.30 (3, 77)	.002^a^	.17	.54 (3, 77)	.655	.02
	Laterality	.55 (1, 79)	.463	.01	57.59 (1, 79)	<.001^a^	.42
	Laterality × group	.04 (1, 79)	.842	.00	2.05 (1, 79)	.156	.03
	Nuclei × laterality	2.21 (3, 77)	.094	.08	4.82 (3, 77)	.004^a^	.16
	Nuclei × laterality × group	.81 (3, 77)	.492	.03	.18 (3, 77)	.910	.01
	Group	.001 (1, 79)	.978	.00	.13 (1, 79)	.721	.00
With vs. without history of FBTCS	Nuclei	4.16 (3, 125)	.008^a^	.09	22.11 (3, 125)	<.001^a^	.35
	Nuclei × group	.38 (3, 125)	.766	.01	2.75 (3, 125)	.046^a^	.06
	Laterality	.43 (1, 127)	.515	.00	52.55 (1, 127)	<.001^a^	.29
	Laterality × group	.05 (1, 127)	.830	.00	.05 (1, 127)	.821	.00
	Nuclei × laterality	2.95 (3, 125)	.035^a^	.07	3.08 (3, 125)	.030^a^	.07
	Nuclei × laterality × group	.77 (3, 125)	.513	.02	1.15 (3, 125)	.332	.03
	Group	.91 (1, 127)	.342	.01	.06 (1, 127)	.808	.00
With vs. without history of status epilepticus	Nuclei	2.78 (3, 131)	.044^a^	.06	17.99 (3, 131)	<.001^a^	.29
	Nuclei × group	.70 (3, 131)	.551	.02	1.27 (3, 131)	.289	.03
	Laterality	1.75 (1, 133)	.188	.01	54.06 (1, 133)	<.001^a^	.29
	Laterality × group	.94 (1, 133)	.335	.01	1.62 (1, 133)	.206	.01
	Nuclei × laterality	2.43 (3, 131)	.068	.05	1.82 (3, 131)	.147	.04
	Nuclei × laterality × group	.26 (3, 131)	.853	.01	.50 (3, 131)	.686	.01
	Group	.89 (1, 133)	.346	.01	2.59 (1, 133)	.110	.02
Congenital vs. acquired etiologies	Nuclei	1.17 (3, 114)	.325	.30	16.49 (3, 114)	<.001^a^	.30
	Nuclei × group	.79 (3, 114)	.501	.20	.54 (3, 114)	.653	.01
	Laterality	.30 (1, 116)	.863	.00	28.29 (1, 116)	<.001^a^	.20
	Laterality × group	.18 (1, 116)	.671	.00	5.46 (1, 116)	.021^a^	.05
	Nuclei × laterality	1.40 (3, 114)	.246	.36	1.87 (3, 114)	.139	.05
	Nuclei × laterality × group	.43 (3, 114)	.734	.01	.96 (3, 114)	.415	.03
	Group	.26 (1, 116)	.611	.00	1.94 (1, 116)	.166	.02
Postsurgically seizure‐free vs. not seizure‐free	Nuclei	1.54 (3, 82)	.211	.05	11.17 (3, 82)	<.001^a^	.29
	Nuclei × group	1.07 (3, 82)	.367	.04	.12 (3, 82)	.949	.00
	Laterality	.09 (1, 84)	.756	.00	33.58 (1, 84)	<.001^a^	.29
	Laterality × group	1.16 (1, 84)	.284	.01	.19 (1, 84)	.667	.00
	Nuclei × laterality	3.34 (3, 82)	.023^a^	.11	2.52 (3, 82)	.064	.08
	Nuclei × laterality × group	1.2 (3, 82)	.316	.04	.36 (3, 82)	.780	.01
	Group	.07 (1, 84)	.796	.00	.8 (1, 84)	.375	.01

Abbreviations: FBTCS, focal to bilateral tonic–clonic seizures; FLE, frontal lobe epilepsy; HS, hippocampal sclerosis; PQE, posterior quadrant epilepsy; Sig., significance; TLE, temporal lobe epilepsy; df, degrees of freedom.

^a^ Significant at *p* < .05 or less. Post hoc pairwise comparisons were performed for significant effects to determine the nature of the differences between those variables (Tables S1 and S2).

**TABLE 3:** One‐sample *t*‐tests comparing patient *Z*‐scores to zero (the control mean) and paired *t*‐tests for laterality effects (ipsilateral vs. contralateral).

Group	Nucleus	One‐sample *t*‐tests comparing patient group to zero (the control mean)										Paired *t*‐test results for laterality effects (ipsilateral vs. contralateral)			
		Ipsilateral					Contralateral								
		Mean	SD	*t*	Sig.	Cohen *d*	Mean	SD	*t*	Sig.	Cohen *d*	Mean diff.	*t*	Sig.	Cohen *d*
Overall focal epilepsy, *n* = 136	Node strength														
	Anterior	−.15	1.04	−1.65	.808	.14	−.19	1.01	−2.22	.224	.19	.04	.41	1.000	.04
	Lateral	−.23	1.03	−2.59	.088	.22	−.12	1.16	−1.20	1.000	.10	−.11	−1.24	.876	.11
	Medial	−.08	1.10	−.80	1.000	.07	−.25	1.07	−2.67	.072	.23	.17	1.81	.289	.16
	Pulvinar	−.56	1.01	−6.47	<.001^a^	.55	−.28	1.08	−2.97	.032^a^	.25	−.28	−2.99	.013^a^	.26
	Volume														
	Anterior	−.60	1.19	−5.88	<.001^a^	.50	.01	1.30	.13	1.000	.01	−.62	−6.34	<.001^a^	.54
	Lateral	−.52	1.08	−5.64	<.001^a^	.48	−.12	1.11	−1.29	1.000	.11	−.40	−6.69	<.001^a^	.57
	Medial	.31	1.44	2.47	.117	.21	.51	1.52	3.91	<.001^a^	.33	−.20	−2.63	.038^a^	.23
	Pulvinar	−.09	1.43	−.73	1.000	.06	.24	1.25	2.22	.223	.19	−.33	−3.66	<.001^a^	.31
TLE, *n* = 81	Node strength														
	Anterior	−.20	1.07	−1.68	.775	.19	−.17	1.03	−1.52	1.000	.17	−.03	−.18	1.000	.02
	Lateral	−.25	1.06	−2.11	.306	.23	−.18	1.16	−1.39	1.000	.16	−.07	−.56	1.000	.06
	Medial	−.16	1.12	−1.30	1.000	.14	−.29	.98	−2.68	.072	.30	.13	1.06	1.000	.12
	Pulvinar	−.57	1.11	−4.60	<.001^a^	.51	−.32	.99	−2.90	.039^a^	.32	−.25	−2.20	.124	.24
	Volume														
	Anterior	−.64	1.22	−4.72	<.001^a^	.52	.17	1.22	1.28	1.000	.14	−.81	−6.87	<.001^a^	.76
	Lateral	−.57	1.03	−4.98	<.001^a^	.55	−.12	1.15	−.92	1.000	.10	−.45	−5.62	<.001^a^	.62
	Medial	.26	1.49	1.56	.974	.17	.45	1.52	2.70	.068	.30	−.20	−1.97	.211	.22
	Pulvinar	−.13	1.47	−.82	1.000	.09	.20	1.30	1.35	1.000	.15	−.33	−3.32	.004^a^	.37
FLE, *n* = 36	Node strength														
	Anterior	−.15	.96	−.95	1.000	.16	−.23	1.01	−1.36	1.000	.23	.08	.40	1.000	.07
	Lateral	−.23	1.02	−1.38	1.000	.23	−.05	1.18	−.23	1.000	.04	−.19	−1.56	.513	.26
	Medial	.00	1.13	.01	1.000	.00	−.43	1.18	−2.16	.304	.36	.42	2.36	.095	.39
	Pulvinar	−.57	.83	−4.10	<.001^a^	.68	−.15	1.14	−.78	1.000	.13	−.42	−2.13	.161	.36
	Volume														
	Anterior	−.60	1.09	−3.32	.017^a^	.55	−.32	1.41	−1.35	1.000	.22	−.29	−1.68	.411	.28
	Lateral	−.51	1.08	−2.86	.057	.48	−.16	1.06	−.93	1.000	.16	−.35	−4.20	<.001^a^	.70
	Medial	.23	1.47	.96	1.000	.16	.40	1.42	1.68	.809	.28	−.16	−1.11	1.000	.19
	Pulvinar	.36	1.23	1.74	.722	.29	.26	1.12	1.38	1.000	.23	.10	.84	1.000	.14
PQE, *n* = 19	Node strength														
	Anterior	.09	1.04	.38	1.000	.09	−.19	.93	−.89	1.000	.21	.28	.94	1.000	.22
	Lateral	−.14	1.00	−.61	1.000	.14	.00	1.15	.01	1.000	.00	−.13	−.48	1.000	.11
	Medial	.15	1.00	.64	1.000	.15	.30	1.10	1.18	1.000	.27	−.15	−.69	1.000	.16
	Pulvinar	−.51	.90	−2.45	.200	.56	−.34	1.39	−1.07	1.000	.25	−.17	−.56	1.000	.13
	Volume														
	Anterior	−.43	1.29	−1.45	1.000	.33	−.04	1.31	−.12	1.000	.03	−.39	−1.19	1.000	.27
	Lateral	−.34	1.32	−1.12	1.000	.26	−.07	1.11	−.28	1.000	.06	−.27	−1.32	.812	.30
	Medial	.64	1.19	2.35	.243	.54	.95	1.70	2.43	.206	.56	−.31	−1.36	.756	.31
	Pulvinar	−.75	1.42	−2.31	.264	.53	.39	1.32	1.29	1.000	.30	−1.14	−3.05	.027^a^	.70

*Note*: Probability values are adjusted for multiple comparisons using the Bonferroni method.

Abbreviations: diff., difference; FLE, frontal lobe epilepsy; PQE, posterior quadrant epilepsy; Sig., significance; TLE, temporal lobe epilepsy.

^a^ Significant at *p* < .05.

For volume, a significant group × nucleus interaction was found (*p* < .001, partial η^2^ = .21; Table 2). The main effect of group was not significant (*p* > .05). Specifically, children with focal epilepsy had reduced volumes in the anterior and lateral thalamic nuclei ipsilateral to the seizure focus, and increased volume in the medial nucleus contralaterally (all corrected *p* < .05, Cohen *d* = .33–.50; Figure 2, Table 3). Within patients, all thalamic nuclei had decreased volume on the seizure focus side compared to the contralateral side (all corrected *p* < .05, Cohen *d* = .31–.57).

#### 3.1.2 Subgroup patterns by seizure focus

A significant main effect of nucleus was found for node strength (*p* = .007, partial η^2^ = .09; Table 2), and a group (TLE vs. FLE vs. PQE) × nucleus × laterality interaction for volume (*p* = .001, partial η^2^ = .09), indicating variation in thalamic alterations by seizure focus location and nucleus. Planned post hoc comparisons to controls was performed (Table 3), revealing the following patterns.

##### Temporal lobe epilepsy

Consistent with patterns of the overall focal epilepsy group, children with TLE showed reduced node strength in the pulvinar bilaterally compared to controls (ipsilateral: corrected *p* < .001, Cohen *d* = .51; contralateral: corrected *p* = .039, Cohen *d* = .32; Figure 2, Table 3). Within the TLE group, anterior nucleus connectivity varied by pathology (a significant group × nucleus interaction *p* = .002, partial η^2^ = .17; Figure 2, Table 2). TLE with HS exhibited increased node strength in the bilateral anterior nucleus compared to those without HS (TLE No HS; Table S1). When the anterior nucleus of each subgroup was compared to controls, the TLE No HS group showed bilateral reduction (ipsilateral: *p* = .007, Cohen *d* = .39; contralateral: *p* = .004, Cohen *d* = .42), whereas the TLE‐HS group did not differ significantly from controls (all *p* > .05).

For volume, children with TLE showed significant reductions in the anterior and lateral nuclei ipsilateral to the seizure focus compared to controls (all corrected *p* < .001, Cohen *d* = .52, .55; Figure 2, Table 3). The ipsilateral pulvinar was also reduced relative to the contralateral side (corrected *p* < .01, Cohen *d* = .37). No volume differences were found between the HS and No HS subgroups (all *p* > .05).

##### Frontal lobe epilepsy

Children with FLE exhibited reduced node strength in the ipsilateral pulvinar (corrected *p* < .001, Cohen *d* = .68; Figure 2, Table 3). In terms of volume, the ipsilateral anterior nucleus was significantly smaller than in controls (corrected *p* < .05, Cohen *d* = .55; Figure 2), and the ipsilateral lateral nuclei showed a reduction relative to its contralateral homologue (corrected *p* < .05, Cohen *d* = .70).

##### Posterior quadrant epilepsy

In PQE, alterations were localized to the pulvinar (Figure 2). The ipsilateral pulvinar showed reduced volume relative to the contralateral side (corrected *p* < .05, Cohen *d* = .70; Table 3). Although node strength differences did not reach statistical significance after Bonferroni correction, the effect size suggested a trend of reduction in ipsilateral pulvinar (uncorrected *p* = .025, corrected *p* > .05, Cohen *d* = .56), consistent with findings in the overall focal epilepsy group.

**FIGURE 3:**
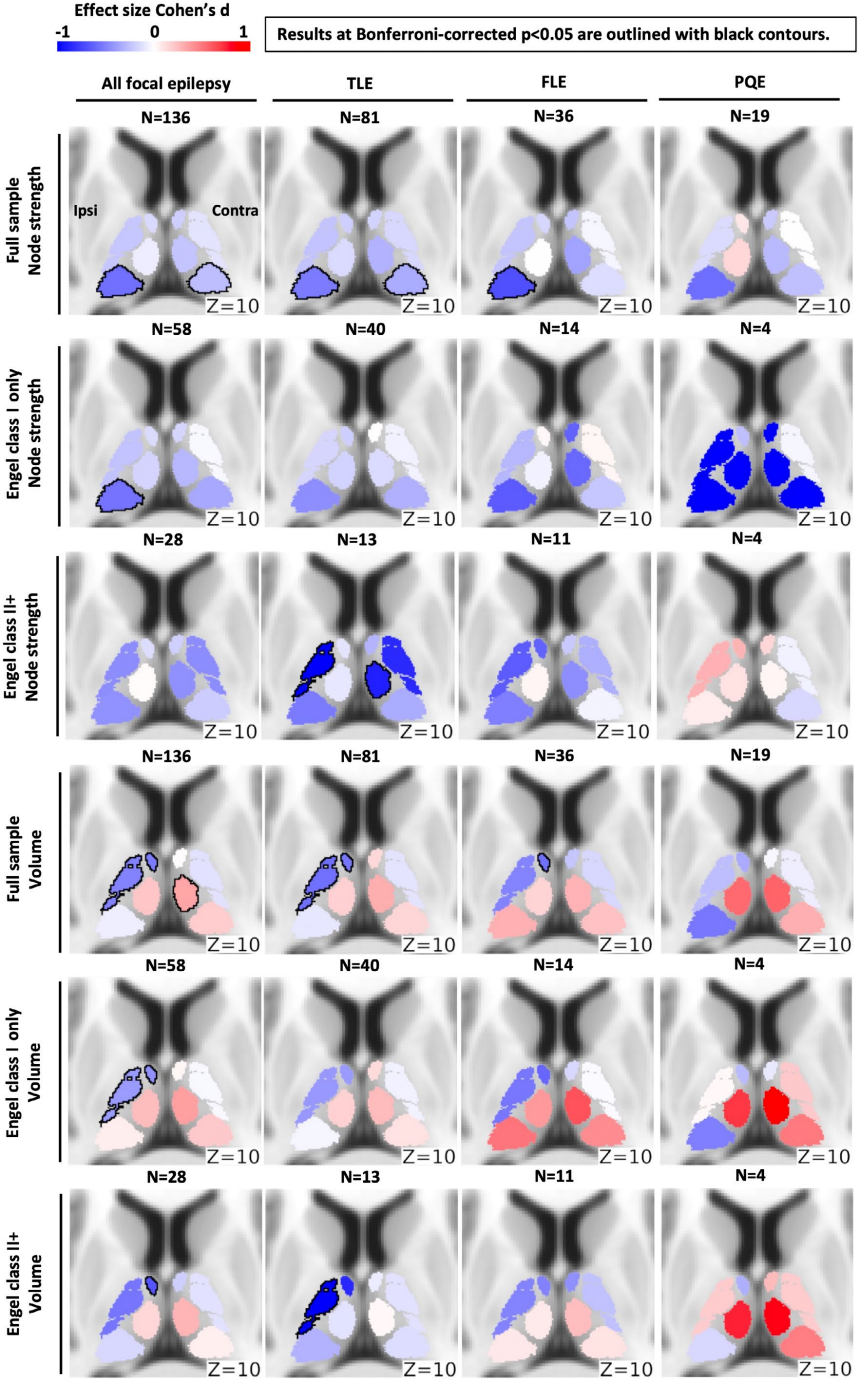
Effect size (Cohen *d*) maps of one‐sample *t*‐tests comparing each epilepsy group (all focal epilepsy, temporal lobe epilepsy [TLE], frontal lobe epilepsy [FLE], posterior quadrant epilepsy [PQE]) against zero (the control mean). Maps are shown for the full cohort, for patients with surgically confirmed epilepsy syndrome who achieved seizure freedom (Engel class I), and for those with postsurgical seizures (Engel class II+). Effect size patterns were qualitatively consistent across groups, particularly for (1) bilateral reductions in functional connectivity, most pronounced in the pulvinar across epilepsy types; and (2) volume loss in most ipsilateral anterior and lateral nuclei, alongside enlargement of contralateral medial nuclei. Effect sizes were slightly attenuated in the FLE and PQE groups, reflecting the instability of Cohen *d* in small samples, where greater variability and lower power can inflate or deflate estimates. Changes in effect size for these smaller subgroups should therefore be interpreted with caution.

Figure 3 shows effect size (Cohen *d*) maps from one‐sample *t*‐tests comparing each epilepsy group (all focal epilepsy, TLE, FLE, PQE) against zero (the control mean) for the full patient cohort, patients with surgically confirmed epilepsy syndrome who achieved seizure freedom (Engel class I), and those with postsurgical seizures.

#### 3.1.3 Comparisons between acquired and congenital etiologies

We grouped patients according to underlying etiology: acquired (*n* = 89, including HS, long‐term epilepsy associated tumors, vascular lesions, and scarring) and congenital (*n* = 29, focal cortical dysplasia). We observed a significant interaction between etiology (acquired vs. congenital) and laterality (ipsilateral vs. contralateral) for thalamic volume (*p* < .05, partial η^2^ = .05; Table 2, Figure 2). Patients with acquired etiologies showed ipsilateral volume loss relative to the contralateral side (pairwise comparison, corrected *p* < .001; Table S3); in contrast, this ipsilateral effect was not significant in the congenital group (corrected *p* = .089).

No significant main effects or interactions involving etiology were found for thalamic node strength (all *p* > .05), suggesting that FC patterns were similar across congenital and acquired groups.

#### 3.1.4 Associations with history of FBTCS

A significant interaction was found between FBTCS history (present vs. absent) and thalamic nucleus for volume (*p* < .05, partial η^2^ = .06; Table 2, Figure 2). Children with focal epilepsy who had experienced FBTCS showed higher volume of the medial nuclei and lower volume of the lateral nuclei, relative to the pulvinar (Table S2). These between‐nucleus volume differences were not observed in patients without FBTCS. In other words, FBTCS history was marked by reduced lateral and increased medial nucleus volumes.

No significant main effects or interactions involving FBTCS history were found for thalamic node strength (all *p* > .05).

#### 3.1.5 Associations with history of status epilepticus

For either thalamic node strength or volume, no significant main effects or interactions involving history of status epilepticus were found (all *p* > .05; Table 2), suggesting that FC and volumetric patterns were similar between patients with and without a history of status epilepticus.

#### 3.1.6 Associations with postsurgical seizure freedom

No significant main effects or interactions related to postsurgical seizure freedom were found on thalamic node strength or volume in the total focal epilepsy sample (all *p* > .05; Table 2).

Exploratory analyses within patient subgroups, including TLE (with and without HS), TLE patients who underwent anterior temporal lobe resection (ATLR), FLE, and PQE, were performed (Table S4). In the TLE No HS group, seizure recurrence was associated with lower bilateral thalamic volume (main effect: *p* = .049, partial η^2^ = .11). No significant differences were observed between seizure‐free and not seizure‐free patients in other subgroups (all *p* > .05).

#### 3.1.7 Correlations of thalamic connectivity, volume, and epilepsy duration

Across the full focal epilepsy cohort, we observed weak negative correlations between epilepsy duration and both connectivity and volume of the lateral thalamic nuclei, yet these associations did not survive correction. In subgroup analyses, longer epilepsy duration was significantly associated with reduced ipsilateral lateral thalamic volume in patients with acquired etiologies (*r* = −.29, corrected *p* = .040) and in the TLE without HS subgroup (*r* = −.39, corrected *p* = .032).

Full correlation matrices and uncorrected *p*‐values are provided in Figure S1.

#### 3.1.8 Edgewise connectivity profiles

**FIGURE 4:**
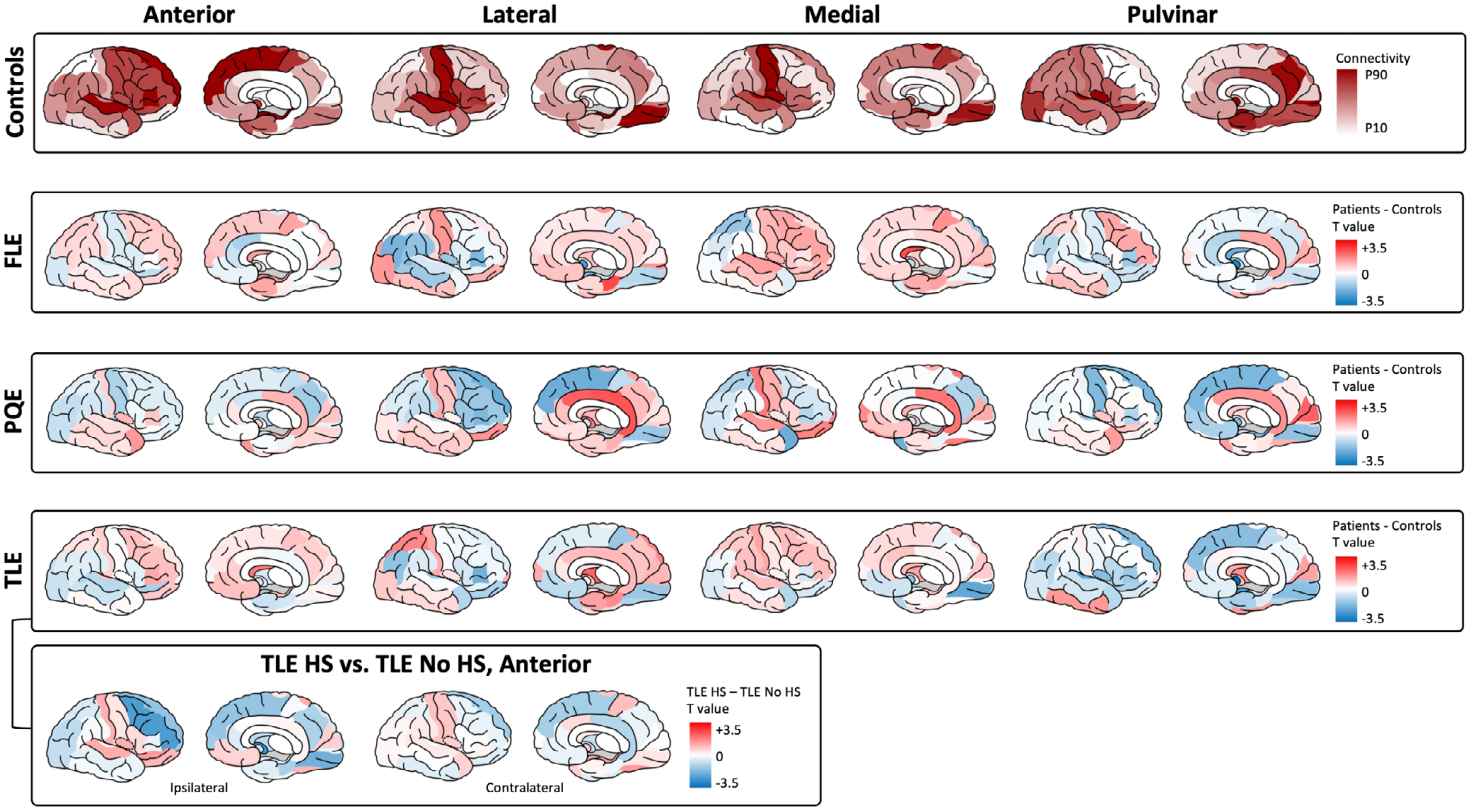
Edgewise connectivity profiles of anterior, lateral, medial, and pulvinar thalamic subdivisions in healthy controls and children with focal epilepsies. The top row displays thalamic connectivity profiles in controls, adjusted for age and sex and averaged across hemispheres. The color scale is set as 10th to 90th percentile of connectivity strength. The lower panels show *t*‐statistics for group differences in connectivity strength between controls and each epilepsy subtype ipsilateral to the seizure focus (contralateral in Figure S2). Red indicates increased connectivity in patients relative to controls (*t* > 0); blue indicates decrease (*t* < 0). For temporal lobe epilepsy (TLE), *t*‐statistics for difference between hippocampal sclerosis (HS) and No HS groups are provided. For exploratory purposes, raw *t*‐values are presented without correction for multiple comparisons. All data are visualized on a right hemisphere template for consistency. FLE, frontal lobe epilepsy; PQE, posterior quadrant epilepsy.

To better interpret the node strength findings, we examined edgewise thalamic connectivity (connections between each thalamic nucleus and all other brain regions; Figure 4). In healthy controls, each nucleus exhibited distinct fMRI connectivity patterns. Table S5 shows the strongest 10 connections of each of the four thalamic nuclei groups. The anterior nucleus primarily connected with the amygdala, hippocampus, superior temporal gyrus, and widespread frontal areas, also linking with striatum and paracentral lobule. Both the lateral and medial thalamic nuclei groups showed strong connections with insular, visual (lingual, fusiform), and sensorimotor (paracentral) regions and temporal lobes. Lateral nuclei uniquely connected with inferior frontal regions (pars triangularis, pars opercularis, pars orbitalis). Medial nuclei uniquely connected with the amygdala and nucleus accumbens. The pulvinar displayed dense connectivity not only with anterior mesial temporal structures (similar to the anterior nucleus) but also extended connections throughout the entire mesial temporal lobe. It also showed strong connections to the accumbens, insula, precuneus, occipital, and posterior cingulate areas. In contrast to the anterior nucleus, the pulvinar had weak connectivity with frontal regions.

The patient groups (TLE, FLE, PQE) showed deviations from the controls' connectivity profiles. These exploratory analyses aimed to contextualize our earlier observation of node strength by identifying thalamocortical and thalamosubcortical disruptions. Figure 4 shows a heterogeneous distribution of increased and decreased FC across the brain between patients and controls. Despite some local increases, these did not outweigh the overall reduction in thalamic node strength when considering whole brain connectivity. Overall, *t*‐values were modest, suggesting that differences at the level of individual connections were subtle. As such, these interpretations should be read with caution; consistent with the reduced node strength of the pulvinar observed across epilepsy groups, we found widespread decreases in its connectivity in the frontal cortex, lateral occipital lobe, lingual gyrus, amygdala, and entorhinal cortex. In TLE patients with HS, the anterior nucleus showed increased node strength compared to those without HS. This difference may be explained by stronger anterior nucleus connectivity with bilateral paracentral regions, the ipsilateral medial orbitofrontal cortex, and contralateral mesial temporal structures.

## 4 DISCUSSION

In this multimodal MRI study, we identified distinct patterns of thalamic FC and volume alterations across childhood focal epilepsy syndromes, compared to typically developing controls.

### 4.1 Thalamic nucleus alterations in focal epilepsies

A major strength of this study is the large pediatric cohort, which enabled comprehensive comparisons across seizure onset locations and pathologies, overcoming the prior focus on adults with mesial TLE.

### 4.2 Functional connectivity

Understanding how the thalamus shapes the growth of seizure networks across the lifespan is a complex but essential endeavor. Here in children, across TLE, FLE, or PQE groups, we observed widespread reductions in thalamic FC, most prominently in the pulvinar. This replicates our prior meta‐analysis reporting thalamic reductions as one of the few consistent FC findings in pediatric epilepsy.^20^ The shared pulvinar hypoconnectivity cannot be explained solely by direct anatomical projections to seizure onset cortices. It also points toward emerging evidence for a "seizure‐suppression" mechanism; increased basal ganglia inhibition of the anterior thalamus,^21^, ^38^ together with reduced coupling between cortical regions^39^ and the posterior thalamus,^40^ may help suppress seizure activity. However, because the thalamus also orchestrates sensory relay, attention, working memory, and executive control,^41^ this protective thalamocortical decoupling may also constrain information processing, contributing to cognitive impairments seen in children with epilepsy.

Connectivity patterns further diverged within TLE depending on pathology. Children with HS showed increased node strength in the anteroventral nucleus, consistent with adult studies linking the anterior thalamus (e.g., increased bilateral thalamohippocampal/limbic FC^42^) to mesial temporal pathologies. Such increases may indicate pathological hyperexcitability along hippocampal–anterior thalamus–cortical circuits. In contrast, TLE without HS exhibited reduced connectivity in the anteroventral nucleus and pulvinar. Seizures from lateral temporal regions can spread rapidly to the pulvinar.^43^ These findings indicate that TLE with and without HS engages thalamocortical networks differently.

We did not observe associations of postsurgical seizure freedom with FC in any nucleus or the entire thalamus in our pediatric cohort. In adults with TLE, He et al.^8^ found that higher FC (measured by degree and eigenvector centrality) of the entire thalamus predicted seizure recurrence after ATLR. They proposed that greater "thalamic hubness" could be a marker of extended seizure propagation networks and a predictor for postoperative seizure recurrence risk. We could not replicate this, likely due to smaller subgroup size, shorter disease duration, or differences in connectivity metrics: He et al. used binary measures (presence or absence of a connection), whereas our node strength additionally considered connection weight and sign (positive or negative). To address this difference, we examined a subsample of children who underwent ATLR (29 seizure‐free, eight not seizure‐free) using the same metric as He et al. but still could not replicate these associations. As noted earlier, adult evidence on nucleus‐specific connectivity and its relationship to surgical outcomes outside TLE and ATLR is limited, making direct comparison with pediatric findings difficult.

### 4.3 Volume

Thalamic volume loss was lateralized to the seizure onset hemisphere, primarily affecting anterior and lateral nuclei in TLE and FLE, consistent with prior studies in adults.^44^, ^45^ Children with PQE showed reduced pulvinar volume ipsilateral to the seizure focus, in line with anatomical pathways from the pulvinar to parietal and occipital cortices (where the PQE seizures have onset).

Compared with the bilateral thalamic atrophy commonly reported in adults,^44^ children in this study showed predominantly ipsilateral reductions. This pattern likely reflects the shorter disease duration in pediatric epilepsy and the mix of congenital and acquired etiologies in our cohort. Supporting this, congenital etiologies showed more diffuse, bilateral thalamic abnormalities, whereas acquired etiologies showed more focal, ipsilateral loss, along with progressive volume reduction with longer epilepsy duration. Combining our pediatric findings with the evidence in adult cohorts allows a tentative conclusion of progressive thalamic atrophy, particularly in acquired forms of epilepsy.

In TLE without HS, lower bilateral thalamic volume was associated with postsurgical seizure recurrence, consistent with adult literature.^10^ However, we did not observe such association in children with TLE‐HS or in the overall TLE group. An unexpected finding was contralateral medial thalamic enlargement in children with a history of FBTCS. This has not been reported previously in focal epilepsy. Acute thalamic swelling has been described shortly after generalized seizures,^46^ and thalamic DWI hyperintensity has been observed following prolonged partial status epilepticus.^47^ One study linked larger thalamic volume to higher seizure frequency in focal epilepsy.^48^ We therefore speculate that medial thalamic enlargement may reflect accumulated seizure burden, particularly given the involvement of the centromedian nucleus in mediating generalized epileptiform propagation.^49^ However, the relationship between acute postseizure edema and the volume increase that we observed here in chronic epilepsy remains unclear. The contralateral increase also poses intriguing questions regarding the potential compensatory brain reorganization in response to seizures.

### 4.4 Limitations and future directions

This study has several limitations. The spatial resolution of fMRI did not allow us to distinguish smaller thalamic nuclei (e.g., the centromedian nucleus within the medial group). Different scanning protocols over the long recruitment period introduced variability, although we used ComBat harmonization to reduce these effects. Some subgroups were small (e.g., patients with PQE, those with unfavorable surgical outcomes), limiting statistical power. We removed task‐related fMRI signals to focus on intrinsic connectivity, but future studies could examine task‐evoked responses to better understand how the thalamus modulates cognitive processes.^2^ Such work may help characterize thalamus‐driven synchronization and its role in directing corticocortical information flow.

Functional and volumetric patterns did not fully overlap, suggesting that each modality reflects distinct aspects of thalamic dysfunction. Future studies combining diffusion MRI, EEG‐derived, and fluorodeoxyglucose PET metrics may provide a more comprehensive understanding of thalamic contributions to seizure networks and surgical outcomes.

## 5 CONCLUSIONS

This study provides novel insights into thalamic structural and functional alterations in pediatric focal epilepsies, characterized by bilateral reductions in FC and ipsilateral volume loss. Patterns of thalamic nuclear involvement varied by seizure focus, reflecting systematic network disruptions with potential implications for personalized interventions such as DBS.

## SOCIAL MEDIA

Multimodal magnetic resonance imaging reveals shared and syndrome‐specific thalamic network alterations across pediatric focal epilepsies. @Feng_Xi_Yu @DNP_ICH #PaediatricEpilepsy #Thalamus #fMRI #MRI #FunctionalConnectivity.

## AUTHOR CONTRIBUTIONS

Xiyu Feng, Torsten Baldeweg, and Rory J. Piper conceived and designed the study. Maria H. Eriksson, Xiyu Feng, Torsten Baldeweg, and Freya Prentice retrieved, anonymized, curated, and verified the data. Xiyu Feng, Rory J. Piper, and Torsten Baldeweg analyzed the data. Xiyu Feng, Rory J. Piper, Martin M. Tisdall, Aswin Chari, and Torsten Baldeweg interpreted the results. Xiyu Feng, Rory J. Piper, and Torsten Baldeweg contributed to drafting the text or preparing the figures. Xiyu Feng, Aswin Chari, Maria H. Eriksson, Freya Prentice, Xiaosong He, Hua Xie, Leigh Sepeta, M. Zubair Tahir, Jonathan D. Clayden, Martin M. Tisdall, Torsten Baldeweg and Rory J. Piper contributed to the writing and approved the final draft of the manuscript.

## CONFLICT OF INTEREST STATEMENT

None of the authors has any conflict of interest to disclose. We confirm that we have read the Journal's position on issues involved in ethical publication and affirm that this report is consistent with those guidelines.

## Supporting information


Data S1.

## Data Availability

To protect patient anonymity, we are unable to publish or share the imaging data. The metadata supporting the findings of this study are available upon request. The CONN toolbox for fMRI preprocessing is available to download at https://web.conn‐toolbox.org/home. The Brain Connectivity Toolbox for computing node strength is available to download at https://sites.google.com/site/bctnet/. The THOMAS tool for computing volume is available to download at https://github.com/thalamicseg/hipsthomasdocker. Subject‐level structural and functional derivatives and all analysis scripts are available to download at https://github.com/yuifeng113/Thalamus‐in‐Children‐with‐Focal‐Epilepsies‐A‐Multi‐Modal‐Neuroimaging‐Study‐Feng‐et‐al‐2025.git.
