# Supplementary material for: Distinct thalamic functional connectivity and volume patterns across focal epilepsies in children: A multimodal neuroimaging study
Source: Epilepsia. 2026 Jan 28;67(5):2358–76. doi: 10.1002/epi.70109 (PMC13179666; doi:10.1002/epi.70109)

Supplementary material

Supplementary Table 1. Pairwise comparisons post a significant group (HS vs. No HS) × nucleus interaction on thalamic node strength.

Nuclei	Mean Difference (HS – No HS)	Std. Error	Sig.[b]
**Anterior**	**.587***	0.181	**0.002**
Lateral	-0.251	0.225	0.197
Medial	-0.291	0.208	0.165
Pulvinar	-0.028	0.215	0.898

*Significant at $p < 0.05$ or less.
b. Adjustment for multiple comparisons: Bonferroni.

Supplementary Table 2. Pairwise comparisons post a significant group (FBTCS vs. No FBTCS) × nucleus interaction on thalamic volume.

Group	(I) Nuclei	(J) Nuclei	Mean Difference (I-J)	Std. Error	Sig.[b]
FBTCS	Anterior	Lateral	.146	.147	1.000
		**Medial**	**-.881***	.213	**.000**
		Pulvinar	-.420	.203	.246
	Lateral	Anterior	-.146	.147	1.000
		**Medial**	**-1.027***	.154	**.000**
		**Pulvinar**	**-.565***	.162	**.004**
	Medial	**Anterior**	**.881***	.213	**.000**
		**Lateral**	**1.027***	.154	**.000**
		**Pulvinar**	**.462***	.142	**.009**
	Pulvinar	Anterior	.420	.203	.246
		**Lateral**	**.565***	.162	**.004**
		**Medial**	**-.462***	.142	**.009**
No FBTCS	Anterior	Lateral	-.045	.107	1.000
		**Medial**	**-.562***	.156	**.003**
		Pulvinar	-.293	.149	.308
	Lateral	Anterior	.045	.107	1.000
		**Medial**	**-.517***	.112	**.000**
		Pulvinar	-.248	.119	.233
	Medial	**Anterior**	**.562***	.156	**.003**
		**Lateral**	**.517***	.112	**.000**
		Pulvinar	.269	.104	.066
	Pulvinar	Anterior	.293	.149	.308
		Lateral	.248	.119	.233
		Medial	-.269	.104	.066

*Significant at $p < 0.05$ or less.
b. Adjustment for multiple comparisons: Bonferroni.

Supplementary Table 3. Pairwise comparisons post a significant group (congenital vs. acquired aetiologies) × laterality (ipsilateral vs. contralateral) interaction on thalamic volume.

Group	Mean Difference (Ipsilateral-Contralateral)	Std. Error	Sig.[b]
**Acquired aetiologies**	**-.461**	.060	**<.001**
Congenital aetiologies	-.180	.105	0.089

*Significant at $p < 0.05$ or less.
b. Adjustment for multiple comparisons: Bonferroni.

Supplementary Table 4 Mixed ANOVAs examining effects of group (post-surgical seizure-free vs. not seizure-free), nuclei and laterality in patient subsets.

Source	Effect	Node Strength			Volume		
		F(df1,df2)	Sig	Partial η^2	F(df1,df2)	Sig	Partial η^2
Temporal lobe epilepsy (TLE)	**Nuclei**	1.00(3,49)	0.401	0.06	3.35(3,49)	**0.026***	0.17
	Nuclei * Group	0.56(3,49)	0.642	0.03	0.01(3,49)	0.999	0.00
	**Laterality**	0.27(1,51)	0.605	0.01	24.90(1,51)	**<0.001***	0.33
	Laterality * Group	1.44(1,51)	0.236	0.03	0.83(1,51)	0.365	0.02

	**Nuclei * Laterality**	1.87(3,49)	0.147	0.10	4.08(3,49)	**0.012***	0.20
	Nuclei * Laterality * Group	0.97(3,49)	0.413	0.06	1.17(3,49)	0.332	0.07
	Group	0.81(1,51)	0.371	0.02	1.20(1,51)	0.279	0.02
TLE with HS	Nuclei	2.26(3,13)	0.129	0.34	1.51(3,13)	0.257	0.26
	Nuclei * Group	1.35(3,13)	0.301	0.24	0.21(3,13)	0.891	0.05
	**Laterality**	0.02(1,15)	0.893	0.00	9.17(1,15)	**0.008***	0.38
	Laterality * Group	3.30(1,15)	0.089	0.18	0.06(1,15)	0.817	0.00
	Nuclei * Laterality	0.95(3,13)	0.445	0.18	1.25(3,13)	0.334	0.22
	Nuclei * Laterality * Group	2.67(3,13)	0.091	0.38	0.52(3,13)	0.677	0.11
	Group	0.08(1,15)	0.548	0.01	1.71(1,15)	0.211	0.10
TLE without HS	Nuclei	0.72(3,32)	0.977	0.06	0.76(3,32)	0.524	0.07
	Nuclei * Group	0.07(3,32)	0.952	0.01	0.25(3,32)	0.863	0.02
	**Laterality**	1.87(1,34)	0.180	0.05	16.49(1,34)	**<.001***	0.33
	Laterality * Group	0.14(1,34)	0.708	0.00	2.38(1,34)	0.132	0.07
	**Nuclei * Laterality**	0.55(3,32)	0.652	0.05	3.67(3,32)	**0.022***	0.26
	Nuclei * Laterality * Group	0.37(3,32)	0.777	0.03	1.41(3,32)	0.257	0.12
	**Group**	1.08(1,34)	0.305	0.03	4.17(1,34)	**0.049***	0.11
Anterior temporal lobe resection (ATLR)	**Nuclei**	1.46(3,33)	0.244	0.12	3.05(3,33)	**0.042***	0.22
	Nuclei * Group	1.32(3,33)	0.285	0.11	0.17(3,33)	0.918	0.01
	**Laterality**	0.11(1,35)	0.746	0.00	10.46(1,35)	**0.003***	0.23
	Laterality * Group	3.05(1,35)	0.090	0.08	0.21(1,35)	0.651	0.01
	Nuclei * Laterality	1.79(3,33)	0.168	0.14	1.40(3,33)	0.260	0.11
	Nuclei * Laterality * Group	1.21(3,33)	0.322	0.10	0.36(3,33)	0.779	0.03
	Group	0.82(1,35)	0.372	0.02	0.00(1,35)	0.976	0.00
Frontal lobe epilepsy (FLE)	**Nuclei**	0.08(3,21)	0.972	0.01	6.68(3,21)	**0.002***	0.49
	Nuclei * Group	0.59(3,21)	0.631	0.08	0.12(3,21)	0.946	0.02
	**Laterality**	0.06(1,23)	0.808	0.00	5.45(1,23)	**0.029***	0.19
	Laterality * Group	0.66(1,23)	0.425	0.03	0.32(1,23)	0.579	0.01
	**Nuclei * Laterality**	7.69(3,21)	**0.001***	0.52	2.23(3,21)	0.114	0.24
	Nuclei * Laterality * Group	0.83(3,21)	0.490	0.11	0.38(3,21)	0.766	0.05
	Group	0.03(1,23)	0.955	0.00	0.73(1,23)	0.400	0.03
Posterior quadrant epilepsy (PQE)	**Nuclei**	7.39(3,4)	**0.041***	0.85	4.72(3,4)	0.084	0.78
	Nuclei * Group	0.29(3,4)	0.830	0.18	0.68(3,4)	0.607	0.34
	Laterality	0.39(1,6)	0.554	0.06	3.29(1,6)	0.120	0.35
	Laterality * Group	1.87(1,6)	0.220	0.24	0.17(1,6)	0.697	0.03
	Nuclei * Laterality	0.38(3,4)	0.775	0.22	0.93(3,4)	0.505	0.41
	Nuclei * Laterality * Group	3.48(3,4)	0.130	0.72	0.27(3,4)	0.844	0.17
	Group	2.10(1,6)	0.198	0.26	0.21(1,6)	0.660	0.03

*Significant at p < 0.05 or less.

Supplementary Table 5. The strongest 10 edges of each of the four thalamic nuclei group (AV, Lat, Med, Pul) in the control cohort.

**Rank**		**AV strongest edges connect to**		**Lat strongest edges connect to**		**Med strongest edges connect to**		**Pul strongest edges connect to**
1		Amygdala		Insula		Insula		Accumbens
2		Superiortemporal		Transversetemporal		Transversetemporal		Transversetemporal
3		Superiorfrontal		Lingual		Hippocampus		Insula
4		Hippocampus		Fusiform		Precentral		Amygdala
5		Parstriangularis		Hippocampus		Lingual		Pericalcarine
6		Frontalpole		Precentral		Amygdala		Precuneus
7		Accumbens		Superiortemporal		Fusiform		Entorhinal
8		Paracentral		Parstriangularis		Paracentral		Parsorbitalis
9		Parsopercularis		Parsopercularis		Superiortemporal		Lateraloccipital
10		Parsorbitalis		Parsorbitalis		Accumbens		Fusiform

The strongest 10 edges/connections between the thalamus and the rest of the brain are ranked from strongest (rank 1) to weakest (rank 10). AV = anteroventral nucleus; Lat = lateral nuclei group (ventral anterior, ventral lateral anterior/posterior, and ventral posterolateral nuclei); Med = medial nuclei group (centromedian and mediodorsal nuclei); Pul = pulvinar.

Supplementary Table 6. Temporal signal-to-noise ratio (tSNR) across the anterior (Ant), lateral (Lat), medial (Med), pulvinar (Pul) thalamic nuclei group for each acquisition protocol. No systematic tSNR differences were observed between patients and controls scanned using the same protocol, supporting that disease effects were not driven by signal quality. tSNR differed between acquisition protocols. Increases in field strength (1.5T to 3T) and faster TR can sometimes improve tSNR but may also increase physiological noise and susceptibility artifacts (Bennett & Miller, 2010). We have applied Combat harmonisation to remove scanning protocol variability. Anterior nuclei had lower tSNR than other nuclei because of tSNR reductions in smaller brain structures (Caparelli et al., 2019).

**Groups**		**tSNR of left thalamic nuclei, Mean (SD)**				**tSNR of right thalamic nuclei, Mean (SD)**			
		**Ant**	**Lat**	**Med**	**Pul**	**Ant**	**Lat**	**Med**	**Pul**
**Protocol 1: Avanto, 3 mm slice thickness, TE/TR=50/3320 ms**	Patients N=48	34(6)	39(6)	38(6)	42(6)	35(7)	39(6)	40(6)	43(6)
	Controls N=41	33(5)	39(3)	40(3)	42(3)	35(6)	40(3)	41(3)	43(3)
**Protocol 2: Avanto, 3 mm slice thickness, TE/TR=30/2160 ms**	Patients N=55	67(11)	78(5)	80(6)	84(6)	68(10)	79(5)	79(6)	82(6)
	Controls N=29	68(11)	75(6)	77(7)	82(6)	68(10)	76(6)	76(7)	79(6)
**Protocol 3: Prisma, 2.5 mm slice thickness, TE/TR=26/1250 ms**	Patients N=33	47(11)	61(10)	56(10)	60(10)	45(12)	62(11)	57(10)	61(11)

Supplementary Table 7. The number of anti-seizure medications (ASMs) used at the time of scanning, and the total number trialled of participants.

	**TLE (n=81)**	**FLE (n=36)**	**PQE (n=19)**	**Control (n=70)**
**Number of ASM at the time of MRI evaluation**				
0	2	0	0	-
1	17	5	2	-
2	23	10	5	-
3+	8	8	2	-
No information	31	13	10	-
**Total number of ASM trialled**				
0	2	0	0	-
1-2	11	5	1	-
3-4	28	9	3	-
5-6	6	4	2	-
7+	3	5	1	-
No information	31	13	12	-

TLE =temporal lobe epilepsy; FLE =frontal lobe epilepsy; PQE =posterior quadrant epilepsy.

Supplementary Figure 1. Associations between thalamic measures and clinical variables in children with focal epilepsy. (A) Relationship between thalamic node strength and epilepsy duration. (B) Relationship between thalamic volume and epilepsy duration. (C) Correlation between thalamic volume and node strength. Pearson's correlations (*r*) with an uncorrected $p<0.05$ are shown. Thalamic subdivisions include Anterior (anteroventral nucleus), Lateral (ventral anterior, ventral lateral anterior/posterior, and ventral posterolateral nuclei), Medial (centromedian and mediodorsal nuclei), and Pulvinar.

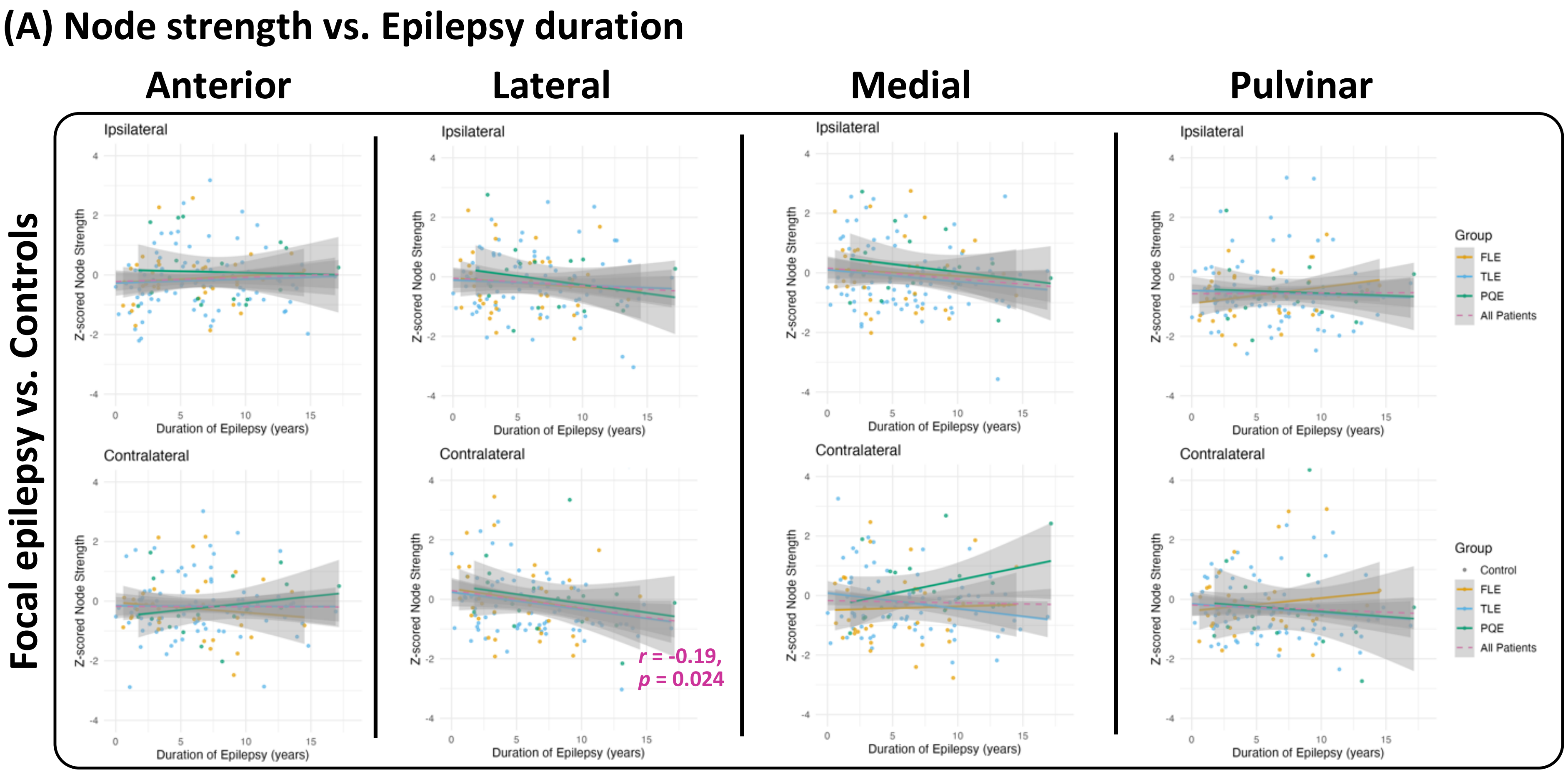

(A) Node strength vs. Epilepsy duration
Anterior
Lateral
Medial
Pulvinar
Focal epilepsy vs. Controls
Ipsilateral
Contralateral
Z-scored Node Strength
Duration of Epilepsy (years)
Group
Control
FLE
TLE
PQE
All Patients
r = -0.19,
p = 0.024

Focal epilepsy with FBTCS

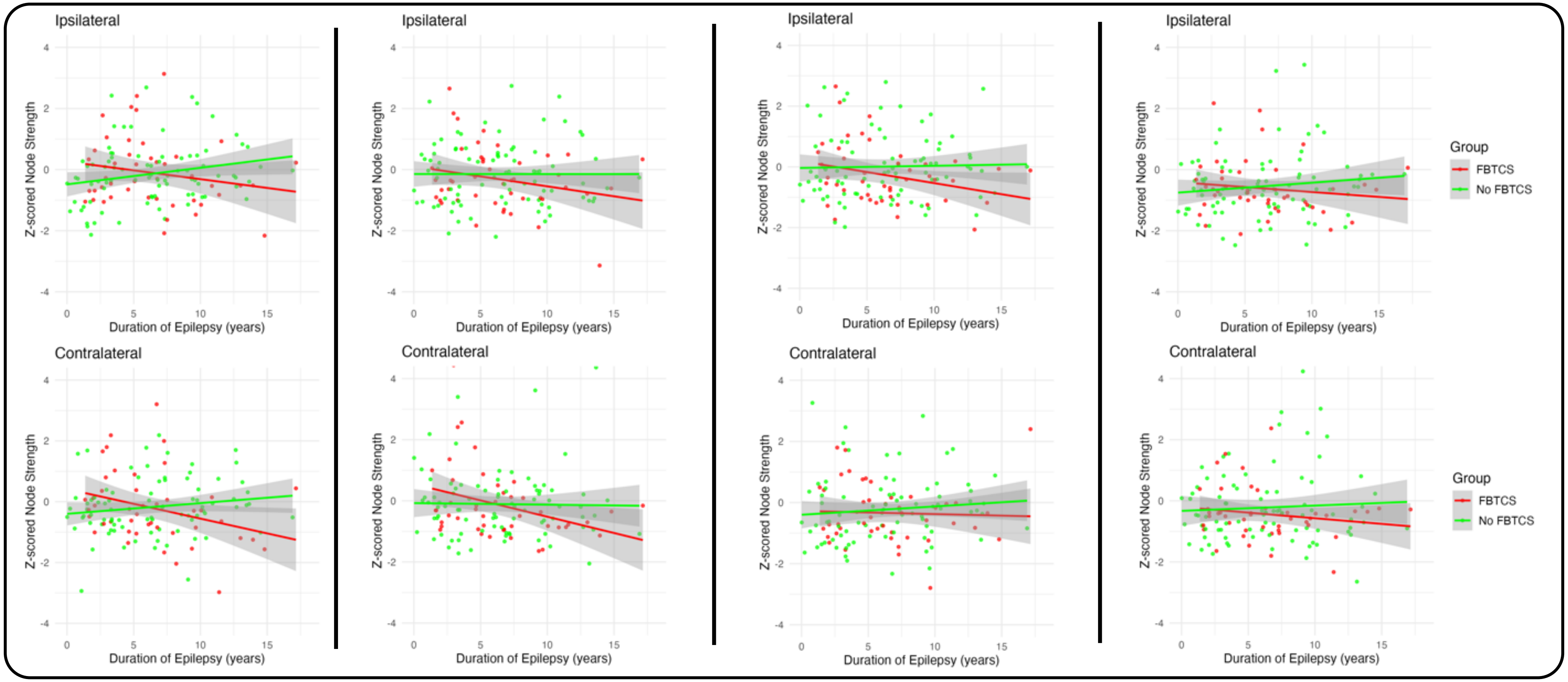

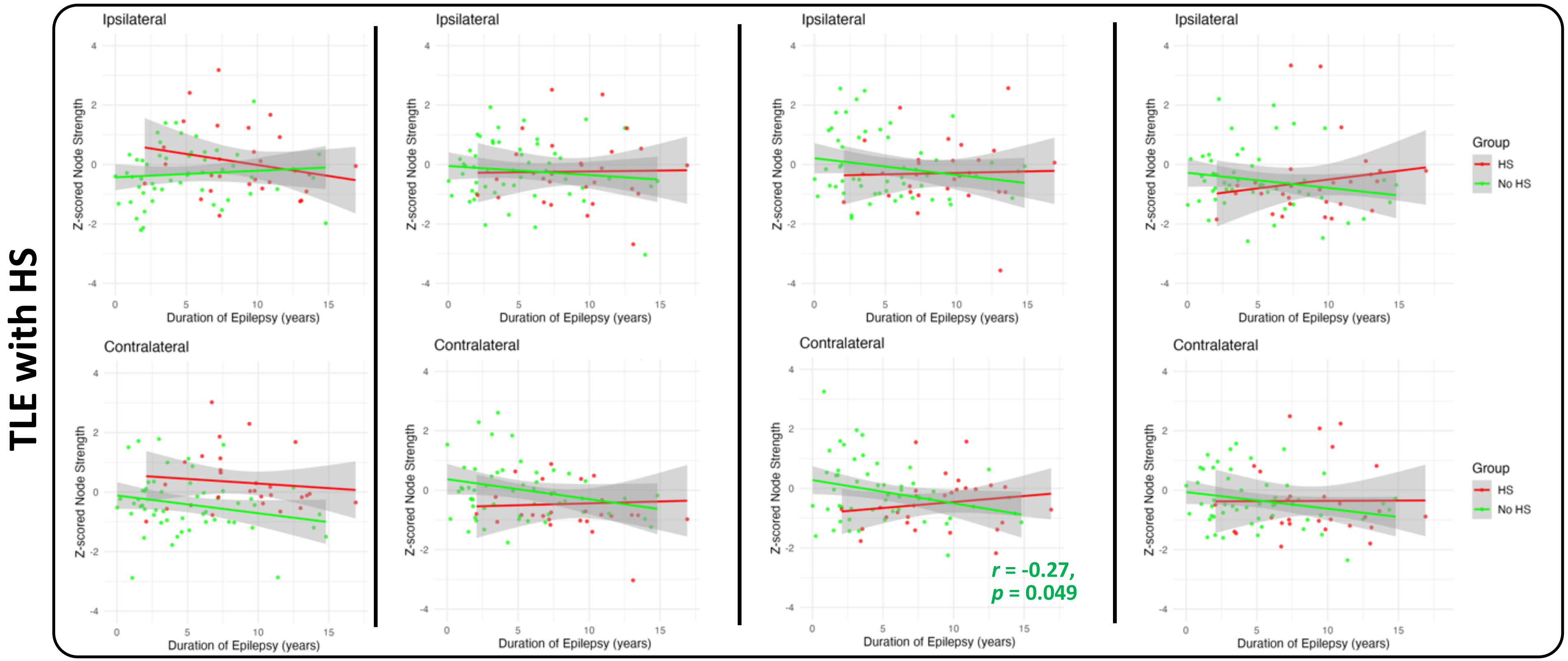

TLE with HS
Ipsilateral
Contralateral
Z-scored Node Strength
Duration of Epilepsy (years)
Group
HS
No HS
r = -0.27,
p = 0.049

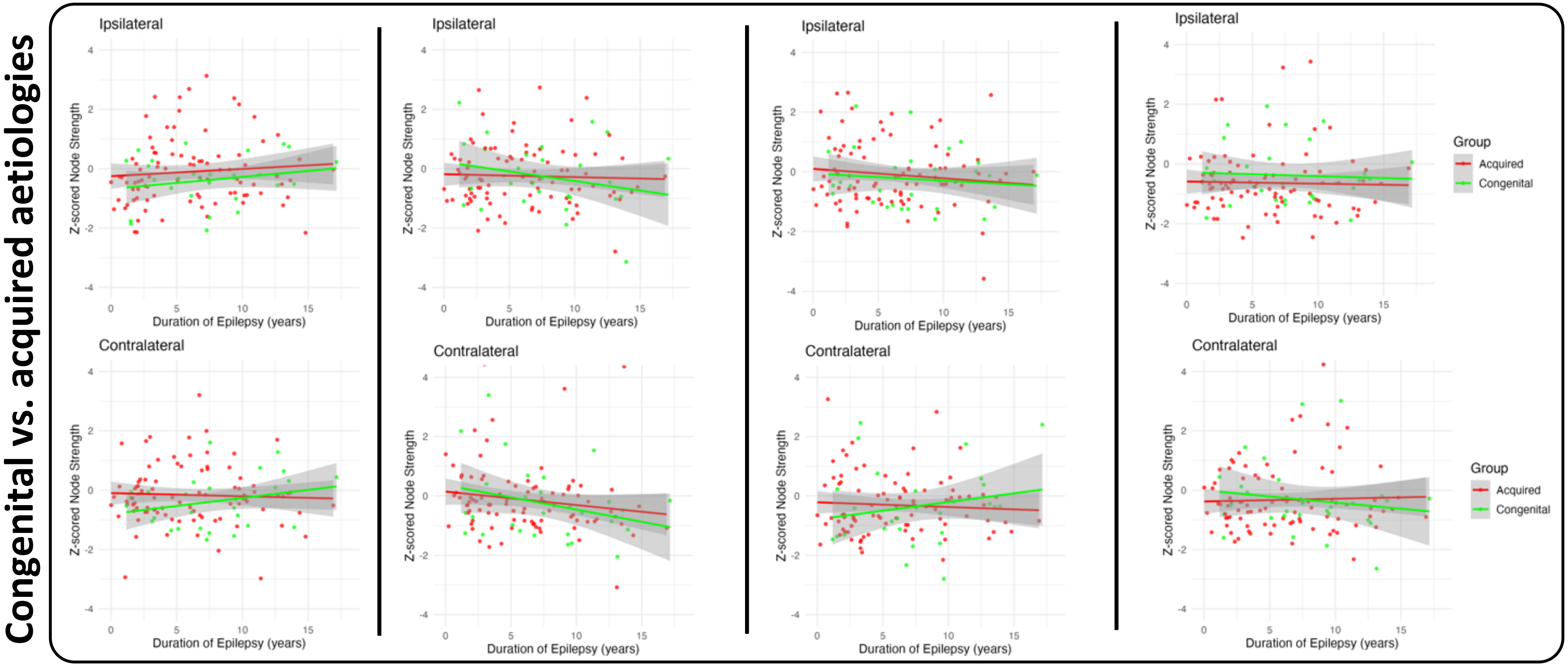
Congenital vs. acquired aetiologies
Ipsilateral
Contralateral
Z-scored Node Strength
Duration of Epilepsy (years)
Group
Acquired
Congenital

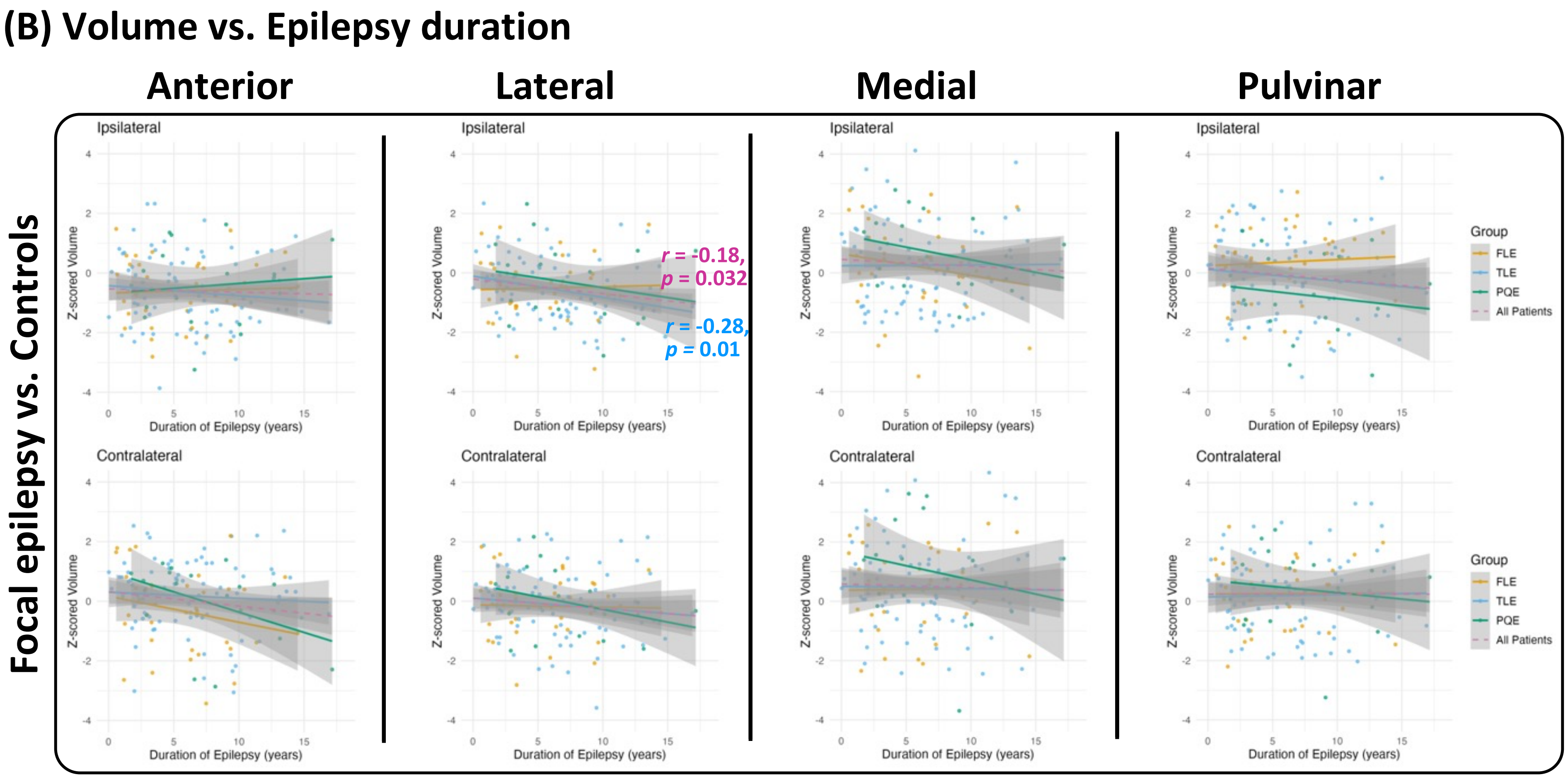
(B) Volume vs. Epilepsy duration
Anterior
Lateral
Medial
Pulvinar
Focal epilepsy vs. Controls
Ipsilateral
Contralateral
Z-scored Volume
Duration of Epilepsy (years)
r = -0.18,
p = 0.032
r = -0.28,
p = 0.01
Group
FLE
TLE
PQE
All Patients

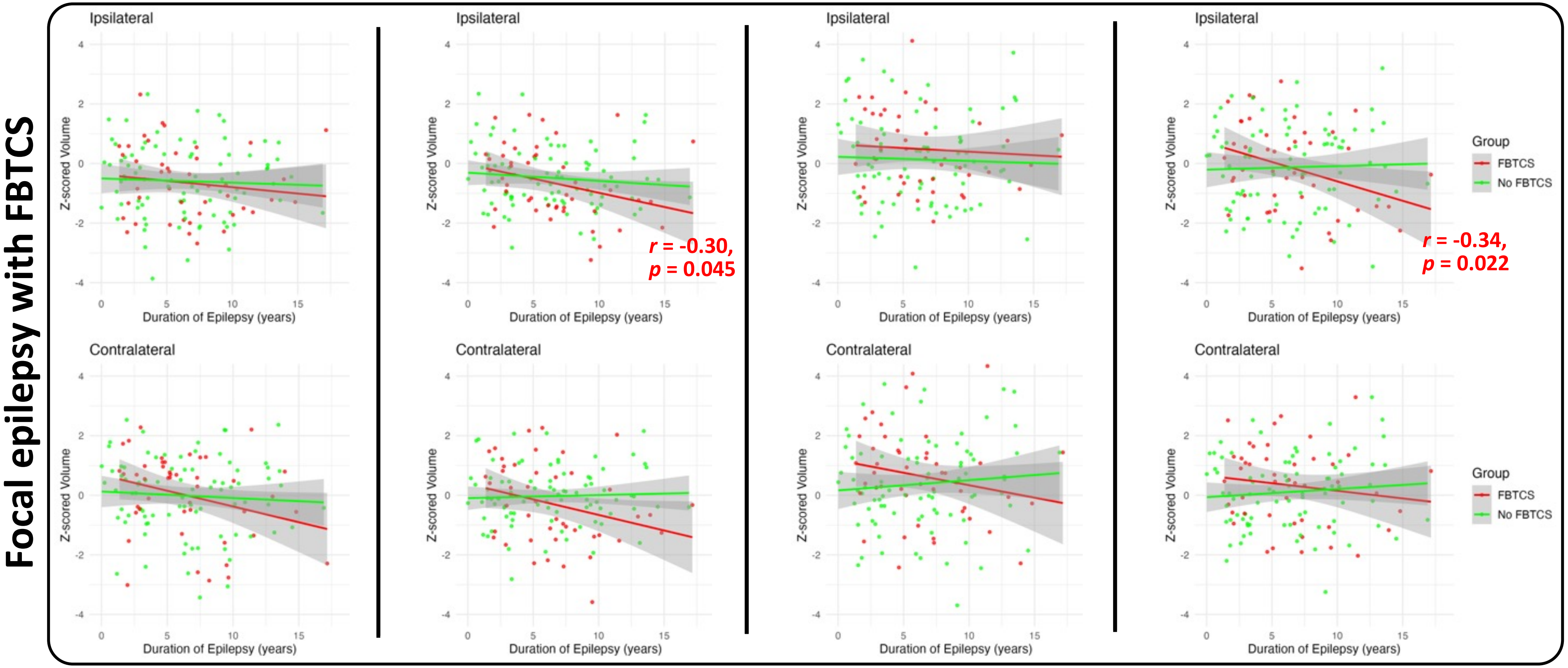
Focal epilepsy with FBTCS
Ipsilateral
Contralateral
Z-scored Volume
Duration of Epilepsy (years)
Group
FBTCS
No FBTCS
r = -0.30,
p = 0.045
r = -0.34,
p = 0.022

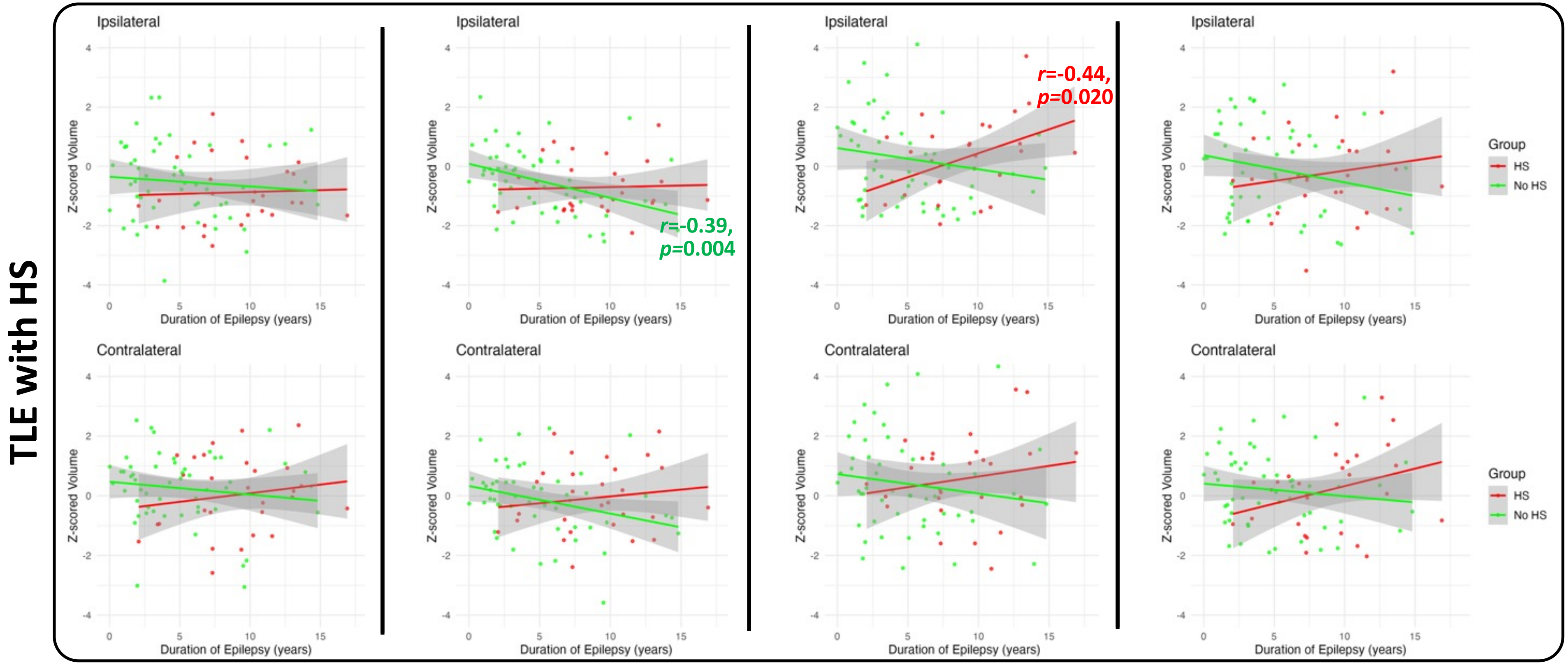
TLE with HS
Ipsilateral
Contralateral
Z-scored Volume
Duration of Epilepsy (years)
r=-0.39,
p=0.004
r=-0.44,
p=0.020
Group
HS
No HS

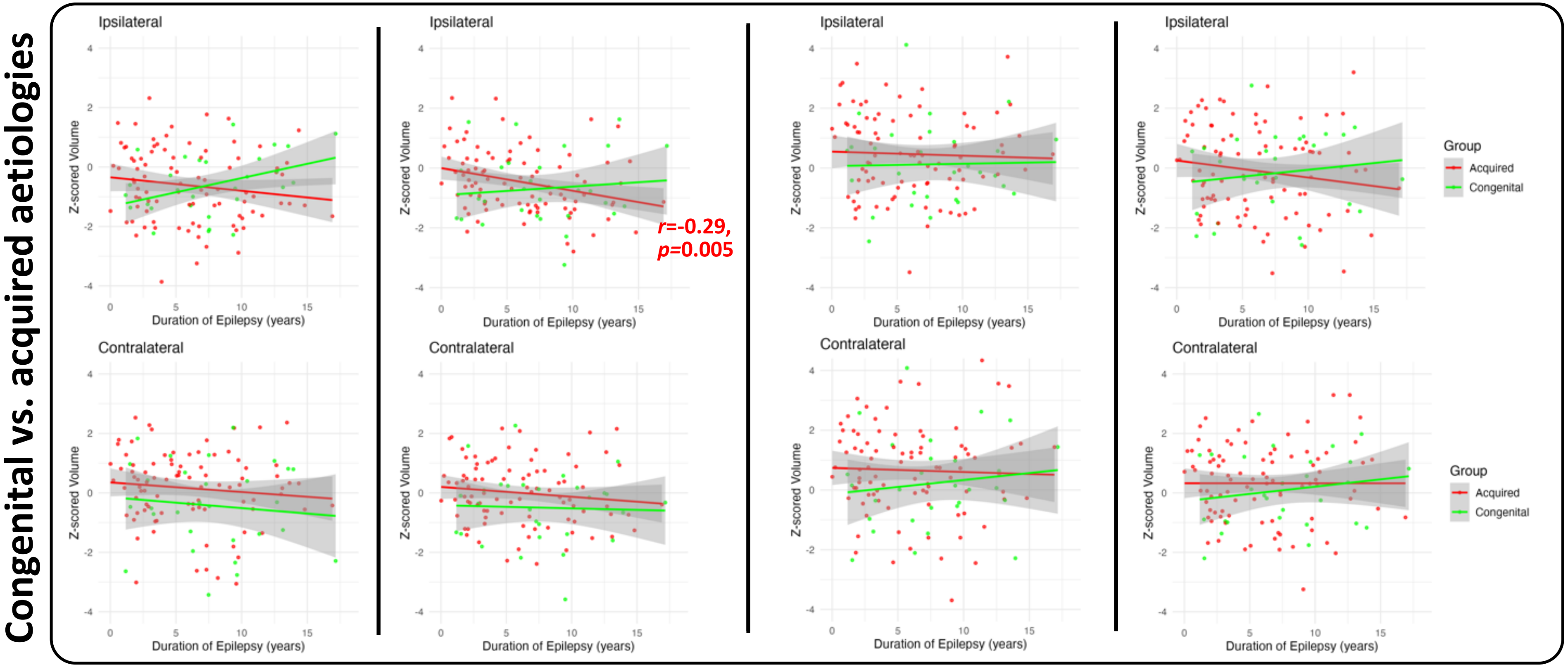
Congenital vs. acquired aetiologies
Ipsilateral
Contralateral
Z-scored Volume
Duration of Epilepsy (years)
r=-0.29,
p=0.005
Group
Acquired
Congenital

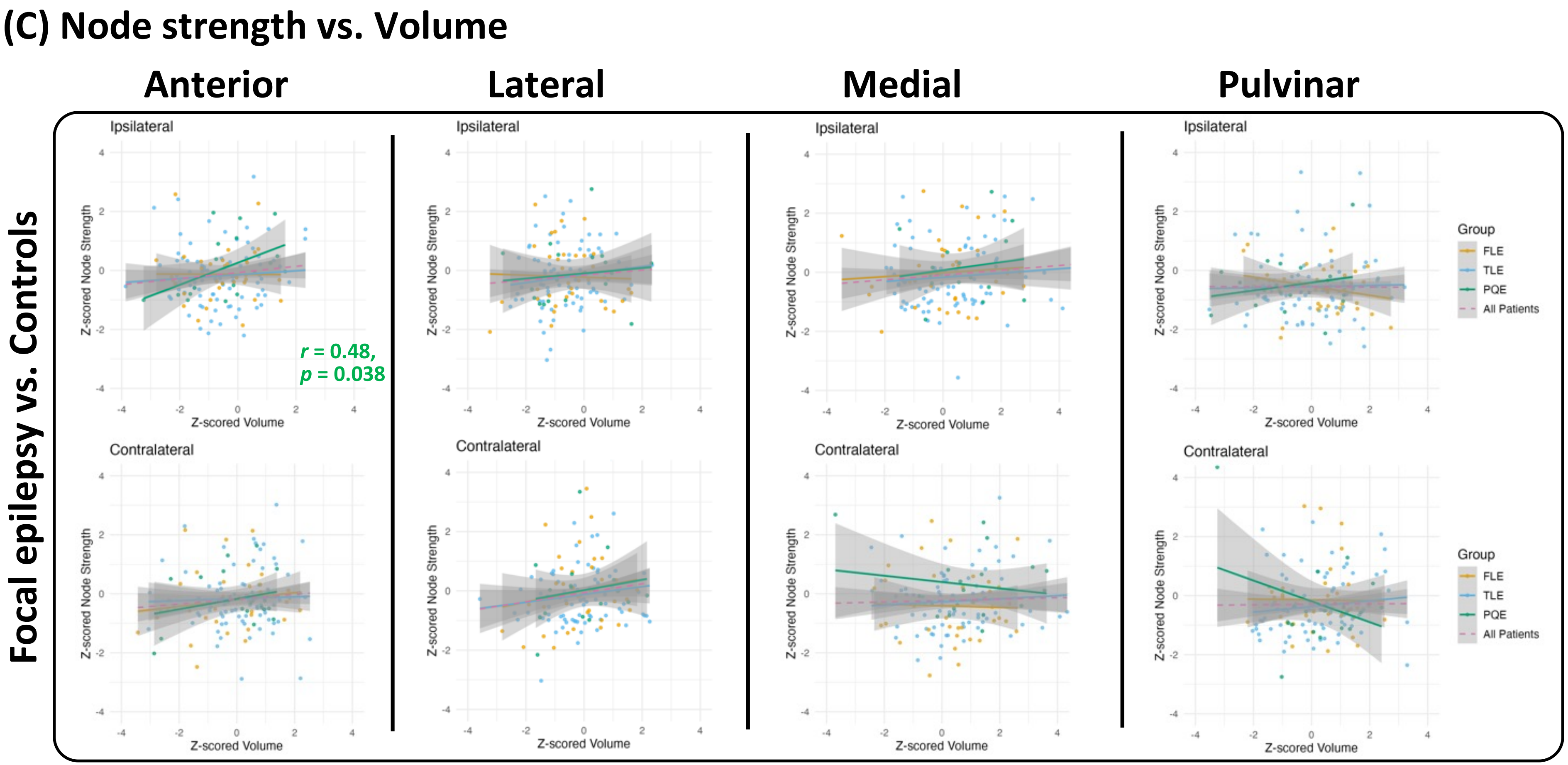
(C) Node strength vs. Volume
Anterior
Lateral
Medial
Pulvinar
Focal epilepsy vs. Controls
Ipsilateral
Contralateral
Z-scored Node Strength
Z-scored Volume
r = 0.48,
p = 0.038
Group
FLE
TLE
PQE
All Patients

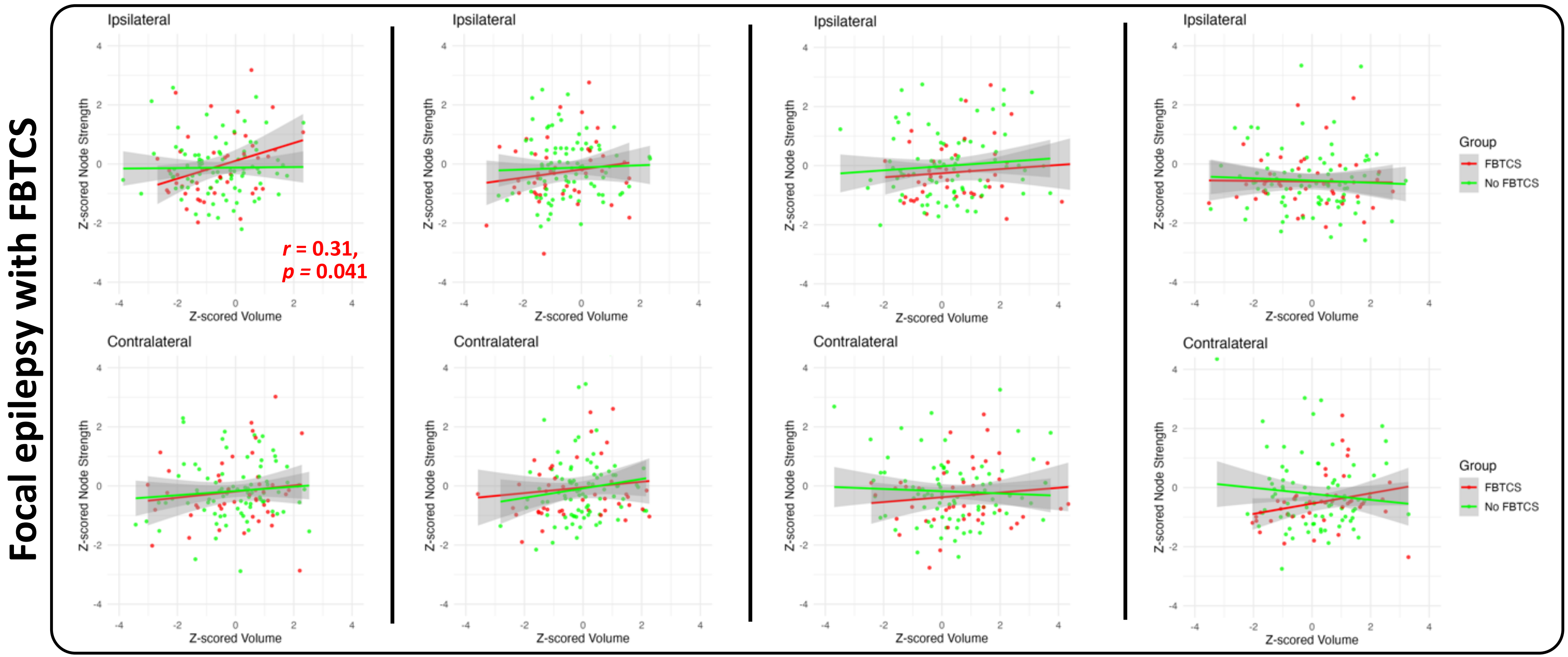
Focal epilepsy with FBTCS
Ipsilateral
Contralateral
Z-scored Node Strength
Z-scored Volume
r = 0.31,
p = 0.041
Group
FBTCS
No FBTCS

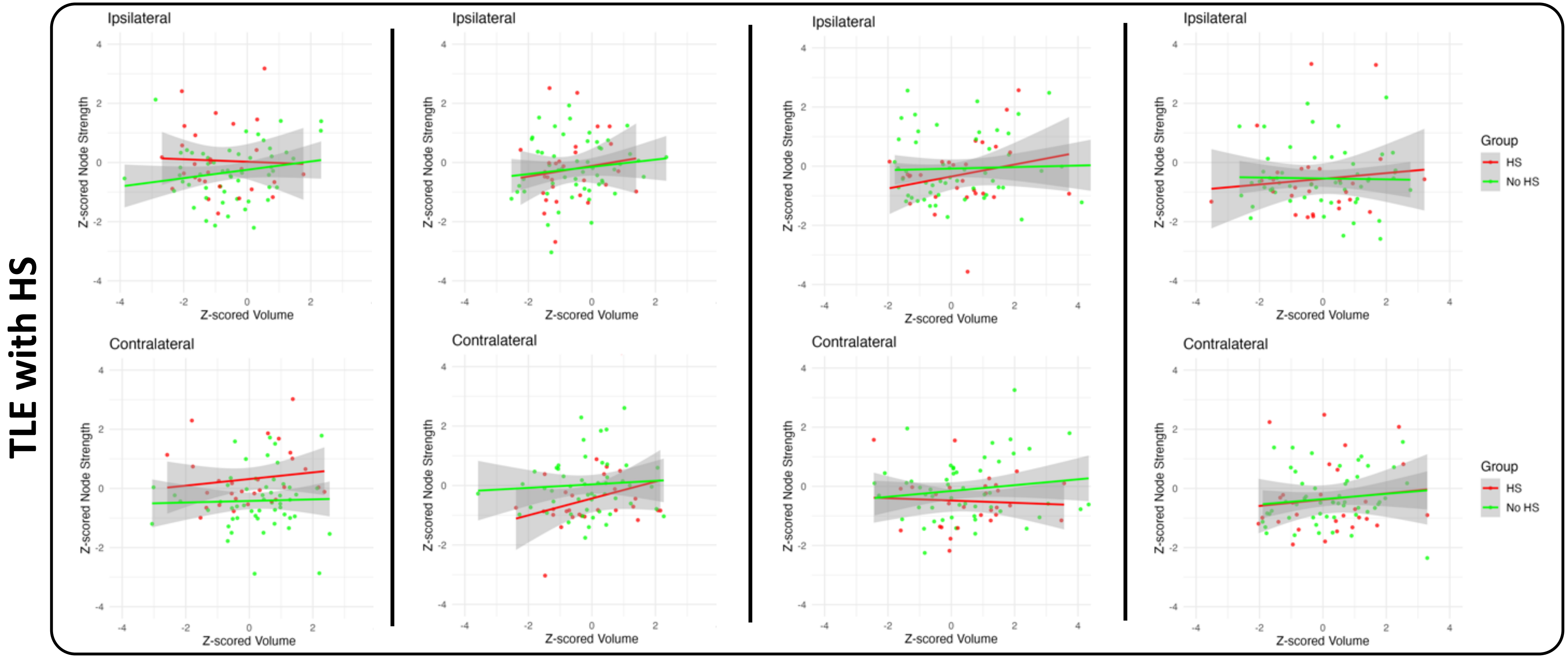
TLE with HS
Ipsilateral
Contralateral
Z-scored Node Strength
Z-scored Volume
Group
HS
No HS

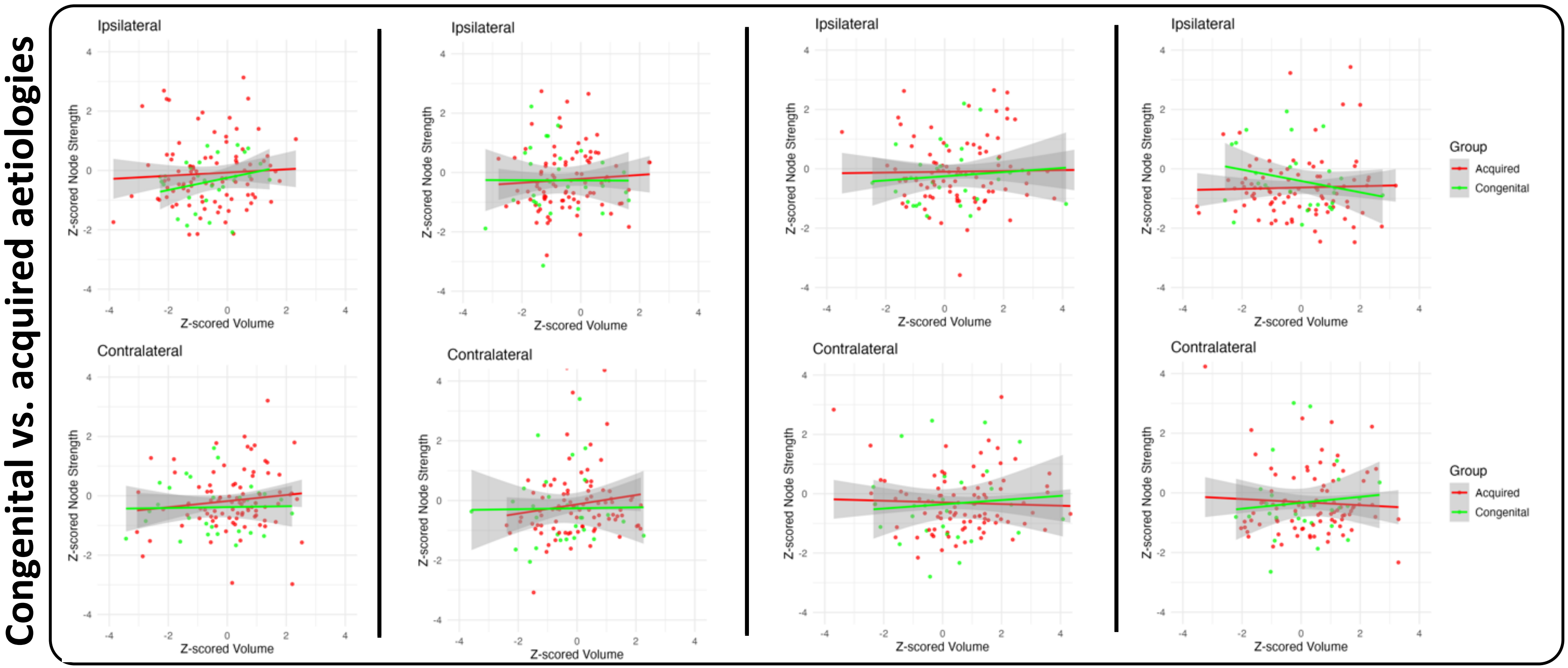
Congenital vs. acquired aetiologies
Ipsilateral
Contralateral
Z-scored Node Strength
Z-scored Volume
Group
Acquired
Congenital

Supplementary Figure 2. Edgewise connectivity profiles of anterior, lateral, medial, and pulvinar thalamic subdivisions in healthy controls and children with focal epilepsies. The top row displays thalamic connectivity profiles in controls, adjusted for age and sex and averaged across hemispheres. The colour scale is set as 10th to 90th percentile of connectivity strength. The lower panels show t-statistics for group differences in connectivity strength between controls and each epilepsy subtype, separately for ipsilateral and contralateral side to the seizure focus. Red indicates increased connectivity in patients relative to controls ($t > 0$); blue indicates decreased connectivity ($t < 0$). For exploratory purposes, raw t values are presented without correction for multiple comparisons. All data are visualised on a right hemisphere template for consistency.

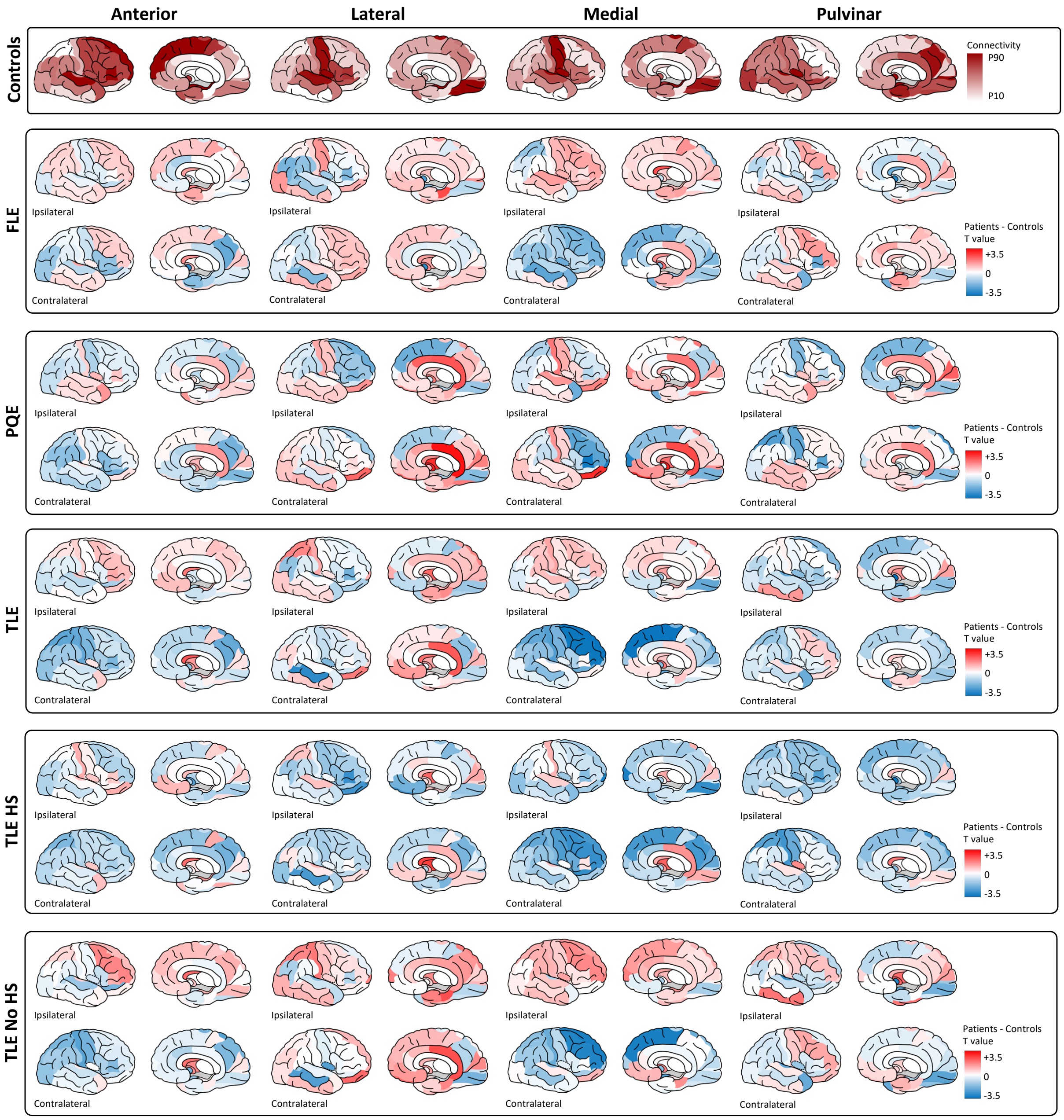
Anterior
Lateral
Medial
Pulvinar
Controls
Connectivity
P90
P10
FLE
PQE
TLE
TLE HS
TLE No HS
Ipsilateral
Contralateral
Patients - Controls
T value
+3.5
0
-3.5

Supplementary Figure 3. A) Data distribution by scanners and harmonization status. B) Area under the receiver operating characteristic curve (AUROC) for random forest classification of scanner strength (1.5T vs. 3T) trained on data before and after ComBat harmonization. ComBat harmonization removes scanner effects that are unrelated to the biological variables while preserving true biological differences in data. After effective harmonization, difference between patients scanned at 1.5T and 3T may persist despite this indicates true biological differences rather than scanner bias. Therefore, instead of directly comparing the two datasets, we evaluated harmonisation performance by training binary classifiers to predict scanner strength, following previous methodological work (Reynolds et al., 2023). High classification accuracy indicates scanner information remained in the data; effective harmonisation should reduce the accuracy to near chance level. For node strength, before- and after-ComBat distributions were nearly identical, with AUROC ≈ 0.5, indicating negligible scanner influence. For volume, ComBat shifted the 1.5 T distribution rightward (green→orange) and the 3 T distribution leftward (purple→pink), increasing their overlap. After ComBat, AUROC dropped significantly (Wilcoxon signed-rank test, $p < 0.001$), indicating scanner-related variance had been removed.

(A) (B)

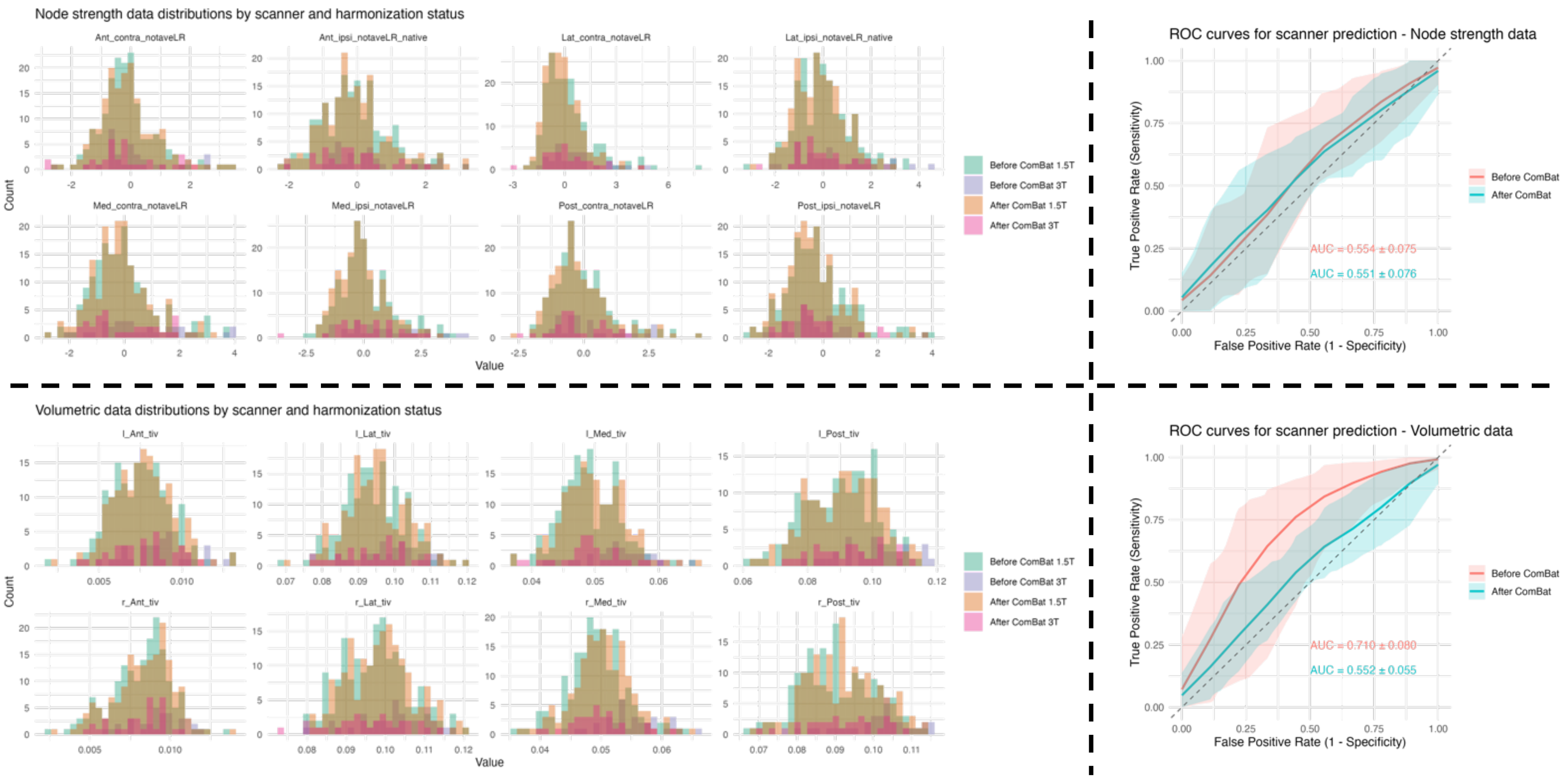
Node strength data distributions by scanner and harmonization status
Ant_contra_notaveLR
Ant_ipsi_notaveLR_native
Lat_contra_notaveLR
Lat_ipsi_notaveLR_native
Med_contra_notaveLR
Med_ipsi_notaveLR
Post_contra_notaveLR
Post_ipsi_notaveLR
Count
Value
Before ComBat 1.5T
Before ComBat 3T
After ComBat 1.5T
After ComBat 3T
ROC curves for scanner prediction - Node strength data
True Positive Rate (Sensitivity)
False Positive Rate (1 - Specificity)
Before ComBat
After ComBat
AUC = 0.554 ± 0.075
AUC = 0.551 ± 0.076
Volumetric data distributions by scanner and harmonization status
l_Ant_tiv
l_Lat_tiv
l_Med_tiv
l_Post_tiv
r_Ant_tiv
r_Lat_tiv
r_Med_tiv
r_Post_tiv
Count
Value
Before ComBat 1.5T
Before ComBat 3T
After ComBat 1.5T
After ComBat 3T
ROC curves for scanner prediction - Volumetric data
True Positive Rate (Sensitivity)
False Positive Rate (1 - Specificity)
Before ComBat
After ComBat
AUC = 0.710 ± 0.080
AUC = 0.552 ± 0.055

Supplementary Figure 4. Effect size (Cohen's d) maps of one-sample t-tests comparing each epilepsy group (all focal epilepsy, TLE, FLE, PQE) against zero (the control mean). Analyses were repeated in (1) the full cohort, and (2) the Avanto-only subsample. Effect size patterns were qualitatively consistent across all analyses, particularly for (a) bilateral reductions in functional connectivity, most pronounced in the pulvinar across epilepsy types, and (b) volume loss in most ipsilateral anterior and lateral nuclei, alongside enlargement of contralateral medial nuclei. These replications indicate that the main findings are robust and not driven by differences in MRI protocols. Effect sizes were slightly attenuated in the FLE and PQE groups, reflecting the instability of Cohen's d in small samples, where greater variability and lower power can inflate or deflate estimates. Changes in effect size for these smaller subgroups should therefore be interpreted with caution.

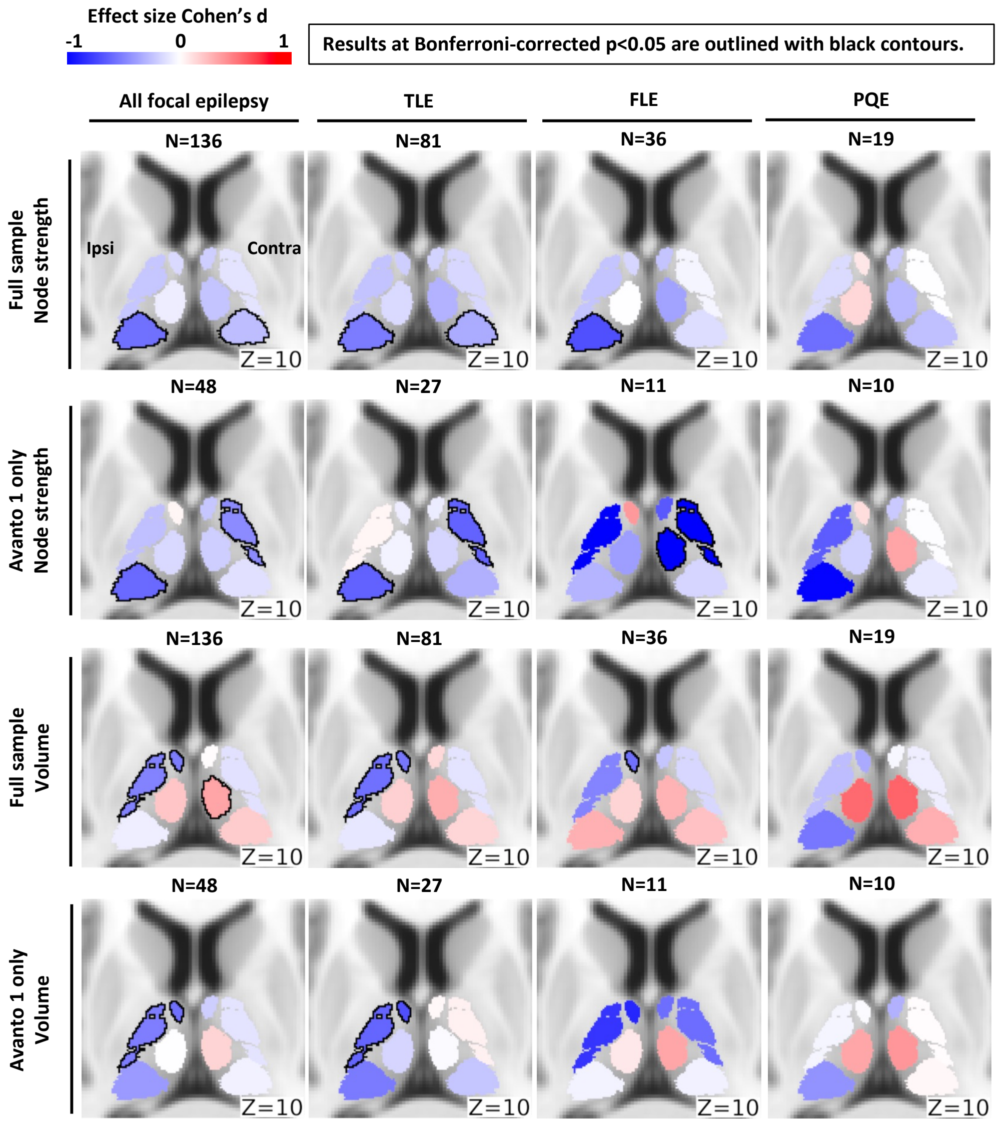

Supplement: Supplementary file 1 — Data S1. [file EPI-67-2358-s001.pdf]
